# HER-2-Targeted Nanoparticles for Breast Cancer Diagnosis and Treatment

**DOI:** 10.3390/cancers14102424

**Published:** 2022-05-13

**Authors:** Leopoldo Sitia, Marta Sevieri, Lorena Signati, Arianna Bonizzi, Arianna Chesi, Francesco Mainini, Fabio Corsi, Serena Mazzucchelli

**Affiliations:** 1 Dipartimento di Scienze Biomediche e Cliniche, Università di Milano, 20157 Milano, Italy; leopoldo.sitia@unimi.it (L.S.); marta.sevieri@unimi.it (M.S.); lorena.signati@unimi.it (L.S.); arianna.bonizzi@unimi.it (A.B.); arianna.chesi@unimi.it (A.C.); francesco.mainini@unimi.it (F.M.); fabio.corsi@unimi.it (F.C.); 2 IRCCS Istituti Clinici Scientifici Salvatore Maugeri, 27100 Pavia, Italy

**Keywords:** nanoparticle, HER-2-positive breast cancer

## Abstract

**Simple Summary:**

Despite tremendous efforts in finding new therapeutic strategies and promoting screening programs to increase early diagnosis, breast cancer is still a major cause of death in the female worldwide population. Preclinical and clinical evidence have shown that nanotechnologies can significantly contribute to improving both therapeutic and diagnostic aspects. This is particularly true for human epidermal growth factor receptor-2 (HER-2) overexpressing (HER-2^+^) breast cancer, where recurrence rates and drug resistance still make it one of the most aggressive breast cancer subtypes, despite the development of promising targeted therapies. The aim of this review is to provide an update on the most promising nanoparticle-based approaches developed in the last decade in the context of HER-2-positive breast cancer therapy and diagnosis.

**Abstract:**

Human epidermal growth factor receptor-2 (HER-2) overexpressing breast cancer is a breast cancer subtype characterized by high aggressiveness, high frequency of brain metastases and poor prognosis. HER-2, a glycoprotein belonging to the ErbB receptor family, is overexpressed on the outer membrane of cancer cells and has been an important therapeutic target for the development of targeted drugs, such as the monoclonal antibodies trastuzumab and pertuzumab. These therapies have been available in clinics for more than twenty years. However, despite the initial enthusiasm, a major issue emerged limiting HER-2 targeted therapy efficacy, i.e., the evolution of drug resistance, which could be tackled by nanotechnology. The aim of this review is to provide a first critical update on the different types of HER-2-targeted nanoparticles that have been proposed in the literature in the last decade for therapeutic purposes. We focus on the different targeting strategies that have been explored, their relative outcomes and current limitations that still need to be improved. Then, we review the nanotools developed as diagnostic kits, focusing on the most recent techniques, which allow accurate quantification of HER-2 levels in tissues, with the aim of promoting more personalized medicinal approaches in patients.

## 1. Introduction

Breast cancer (BC) is the most commonly diagnosed cancer and one of the main causes of death in women, despite advances in early diagnosis and novel therapies [1]. According to GLOBOCAN 2020, BC caused 684,996 deaths (one in six of all cancer deaths) and its incidence was 2.3 million new cases worldwide in 2020 [2]. These numbers highlight that BC still represents a public health problem; therefore it is of paramount importance to identify the most effective therapeutic strategy for the patient. Based on the gene expression profile of biological markers, BC has been classified into four molecular subtypes: Luminal A, Luminal B, HER-2 enriched, and Basal-like[3]. Among them, the human epidermal growth factor receptor 2-positive (HER-2^+^) and HER-2 enriched BC account for 20–30% of BC. These are highly proliferative and aggressive BC subtypes often related to drug resistance [4,5,6,7] and associated with a higher incidence of brain metastasis and worse clinical outcomes [8,9]. From the molecular point of view, they are characterized by the overexpression of HER-2, a glycoprotein with a tyrosine kinase activity, which belongs to the ErbB receptors family. HER-2 is an orphan receptor, which exists as constitutively active form suitable for dimerization with other ErbB members. HER-2-mediated activation of ErbB receptor signaling promotes cell proliferation, motility, differentiation and survival [10]. Hence, HER-2 amplification or over-expression leads to the constitutive activation of the PI3K/Akt and Ras/Raf/MAPK pathways, which results in the development of many epithelial cancers [11,12,13,14]. HER-2 status analysis is performed by immunohistochemistry (IHC) or fluorescence in situ hybridization (FISH), and it has been established that an IHC score of 3+ or 2+, and concomitant amplification, is considered HER-2^+^ BC [15].

Despite a huge effort in research, after the introduction of the first targeted therapies there has been a lack of new agents contributing significantly to HER-2^+^ BC treatment, which is still an unmet clinical need. In our opinion, two factors would greatly contribute to an improvement in HER-2^+^ BC management: the development of new targeted therapeutic strategies and the introduction of new agents allowing a rapid and personalized diagnosis. The aim of this review is to critically highlight the main reasons of this lack of success and to provide an overview of the most promising nanotechnological approaches introduced in the last 10 years on the therapeutic and diagnostic side.

## 2. HER-2^+^ BC Current Therapies and Main Drawbacks 

The first-line treatment for HER-2^+^ BC patients generally includes three steps: first, neoadjuvant chemotherapy is aimed at reducing the tumor size before surgical resection, which consists of a combination of taxanes and dual HER-2 blockade with monoclonal antibodies (mAb) [16,17]. Surgery is then performed to remove the tumor, followed by adjuvant therapy which includes chemotherapy, radiation therapy and targeted therapy to eliminate any remaining tumor cells and to reduce the risk of recurrence [18,19].

The clinical outcomes of the early and metastatic HER-2^+^ patients were extensively improved after the advent of the HER-2 targeted therapies, such as mAb, antibody-drug conjugates, specific pathway inhibitors and immunotherapy. 

### 2.1. Monoclonal Antibodies and Antibody-Drug Conjugates

The first and most relevant example of HER-2^+^-targeted therapy is the FDA approved humanized mAb Trastuzumab (Herceptin^TM^; TZ). TZ has been demonstrated to increase disease-free survival (DFS) and overall survival (OS) in HER-2^+^ BC patients [20,21,22]. Its mechanism of action consists in binding the HER-2 receptor to alter downstream signaling, inhibiting cell cycle progression and consequently arresting tumor growth [23,24]. Despite the wide use of TZ and its success, a great number of patients experience disease progression and relapse due to the onset of several TZ resistance mechanisms such as: (i) heterodimerization with other ErbB members or IGF-1R; (ii) the inactivation of Antibody-Dependent Cellular Cytotoxicity (ADCC); (iii) the expression of the p95-ErbB2 truncated form of the receptor that maintains its intracellular kinase domain; (iv) the decreased levels of expression of the tumor suppressor PTEN, and (v) the hyperactivation of PI3K/AKT pathway [25,26,27,28,29,30]. To limit TZ resistance, a new antibody called Pertuzumab has been developed [19,20,31,32]. Pertuzumab and TZ target two different domains of the ErbB2 receptor (domain II and IV, respectively), and this has proven to prevent heterodimerization with other ErbB receptors, thus limiting a possible cause of resistance. More recently, Margetuximab (Margenza ^®^) has been approved by the FDA based on data from a SOPHIA trial that demonstrated the major advantage of Margetuximab compared with TZ in combination with chemotherapy in pre-treated HER-2^+^ metastatic BC patients [33]. Margetuximab has been associated with a significantly longer PFS and better OS with respect to TZ. As has been shown for other HER-2 inhibitors, one of the side effects that could occur from Margetuximab administration is the left ventricular dysfunction.

An interesting evolution of targeted therapy is the possibility of directly conjugating TZ with anticancer drugs (antibody-drug conjugates, ADCs), thus conferring an intrinsic HER-2 targeting ability to the drug and reducing the toxicity of chemotherapy alone [34]. This approach was exploited to develop Trastuzumab emtansine (T-DM1, Kadcyla^®^, Genentech, San Francisco, CA, USA), one of the commercially available ADCs developed, starting from TZ. The efficacy of T-DM1 in metastatic HER-2^+^ BC was first demonstrated in the EMILIA trial, where improved OS and PFS were observed in patients treated with T-DM1 in comparison to those treated with Capecitabine plus Lapatinib [35]. More recently, the KRISTINE study has demonstrated better efficacy of T-DM1 in the adjuvant setting instead of its use as neoadjuvant therapy [16,19]. In addition to the mechanisms of action of TZ [30,36,37,38], T-DM1 exerts its anticancer activity through internalization in HER-2^+^ cancer cells, where the conjugated drug DM1 prevents the assembly of the mitotic spindle, leading to cell cycle arrest. However, T-DM1 efficacy is still limited by the onset of resistance mechanisms adopted by tumor cells to evade DM1 activity, such as overexpression of drug efflux transporters that excrete DM1 outside BC cell [39,40]. Among the ADC categories, Trastuzumab Deruxtecan (T-Dxd, DS-8201, Enhertu^®^, Daiichi Sankyo, Tokyo, Japan) has recently received accelerated approval by the FDA on the basis of its adequate efficacy and safety demonstrated in the DESTINY-Breast01 study. T-Dxd is recommended for the treatment of patients with unresectable or metastatic HER-2^+^ BC who have already received at least two or more prior anti-HER-2^+^ treatments [41]. The conjugated cytotoxic drug Dxd is a topoisomerase I inhibitor attached to TZ with a tetrapeptide-based linker. It is stable in the plasma but easily cleavable near tumor cells because of the presence of cathepsins highly expressed on their surface. Indeed, the drug has selective permeability of the cell membrane of tumor cells in HER-2^+^ low-expressing cells [42]. Despite the great potential of the response in T-DM1-resistant HER-2^+^ BC, T-Dxd can lead to interstitial lung disease. In fact, adverse reactions caused by its administration are currently under investigation in ongoing clinical trials.

### 2.2. HER-2 Pathway Inhibitors and Immunotherapy

In addition to mAb and ADCs, another HER-2 targeted therapeutic strategy is blockade of the main cellular pathways leading to the characteristic aggressiveness of HER-2^+^ BC, such as PI3K/AKT/mTOR and MAPK pathways, by using specific inhibitors. The aberrant activation of PI3K/mTOR/AKT pathway in BC is mediated by the mutation of the PI3KCA gene (more frequent in ER^+^ and HER-2^+^) [25], which results in its hyperactivation with consequent dysfunction in the PTEN gene, resulting in AKT overactivation. These mutations lead to uncontrolled cell growth, migration and deregulated apoptosis [43,44]. Several examples of PI3K/AKT/mTOR pathway inhibitors have been developed, all interfering with one or more of the pathway’s components. Among these, the most promising ones are: Everolimus, Alpelisib, Taselisib and Buparlisib [45]. Everolimus in combination with Paclitaxel or Vinorelbine and TZ has displayed increased efficacy in metastatic BC patients, although it did not affect PFS [45]. Typically, these inhibitors are used in relapsed BC as a second-line treatment to induce cell death and inhibit cancer cell proliferation. 

Another HER-2-targeted therapy is based on small tyrosine kinase inhibitors (TKIs), such as Neratinib, Lapatinib, Tucatinib and Afatinib [46,47]. These inhibitors are used in advanced or relapsed HER-2^+^ BC as a second-line treatment and are able to inhibit HER-2 receptor kinase activity. Neratinib, which has been validated in combination with Capecitabine in advanced or metastatic HER-2^+^ BC [46,48,49,50], has resulted in a higher rate of pathological complete response (pCR) in comparison to TZ plus chemotherapy [48]. Moreover, Lapatinib in combination with Capecitabine has improved the OS in patients previously treated with TZ and standard chemotherapy [51], although mechanisms of resistance often arise.

Since increased levels of tumor-infiltrating lymphocytes (TILs) in HER-2^+^ BC have been associated with a higher rate of pCR, good prognosis and improved survival after neoadjuvant chemotherapy [52], an immunotherapeutic approach has also been proposed in the last few decades. Indeed, the expression of PD-L1 in HER-2^+^ BC is associated with increased OS [53] and monoclonal antibodies such as, Atezolizumab, Pembrolizumab, Nivolumab, directed against PD-1 receptor and its ligand PD-L1, are able to restore antitumoral immunity [54,55,56].

## 3. Nanotechnological Strategies to Target HER-2 Receptor

Despite the available HER-2 targeted therapies described above, a significant fraction of patients relapse or progress due to the escape mechanisms adopted by HER-2^+^ cancer cells in response to inhibitory therapies. Nanotechnology promises to overcome some of these clinical challenges by the development of novel HER-2-guided nanosystems suitable as powerful tools in cancer imaging, targeting and therapy [57]. During the past 10 years, there has been an increase in the number of scientific publications based on nanotechnological strategies addressing HER-2^+^ BC specifically [58]. Hence, our intention is to propose a comprehensive review concerning the main approaches for therapy and diagnosis to fight HER-2^+^ BC developed in the last decade.

Most engineered NPs developed for HER-2^+^ BC therapy take advantage of HER-2 receptor targeting strategies. Active targeting techniques have been employed by applying ligands to NP surfaces, such as antibodies, peptides and aptamers, that selectively recognize overexpressed receptors on cancer cells. This is aimed at accumulating the nanosystem in the cancer-affected areas, as well as favoring controlled and specific internalization in tumor cells, resulting in enhanced therapeutic efficacy [57]. Achieving specific targeting is so crucial that researchers constantly propose new strategies to drive the HER-2 targeting process. As said, the plethora of proposed solutions is so wide that finding the most robust ones to guide further translational research is an extremely hard task.

**Figure 1:**
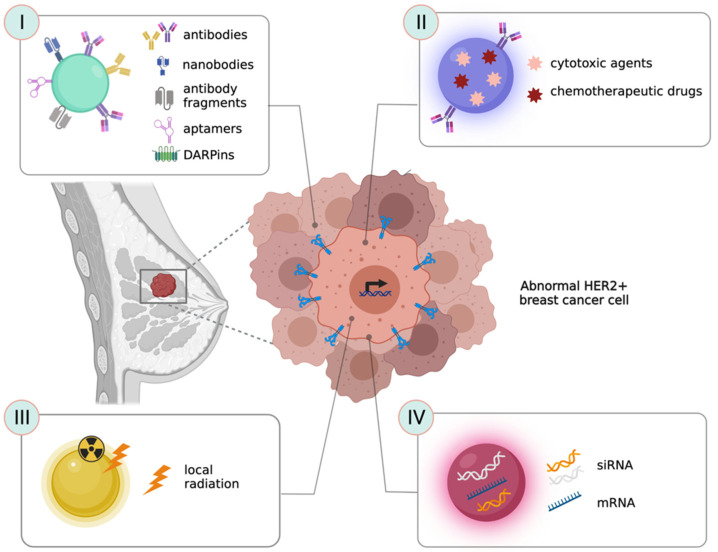
Strategies for HER-2^+^ BC therapy (**I**) NPs functionalized with HER-2 targeting ligands ensure a specific targeting into HER-2^+^ cancer cells; (**II**) NPs can vehicle chemotherapeutic drugs or cytotoxic agents into HER-2^+^ cancer cells; (**III**) NPs functionalized with photothermal agents can promote local radiation to obtain tumor ablation; (**IV**) NPs can deliver nucleic acids or gene silencing molecules to enable gene expression regulation and overcome the insurgence of resistance to conventional therapies.

Here, we describe the most recent and successful strategies used to functionalize NPs with specific HER-2 targeting agents (Figure 1I–IV). As this is strictly related to improving the interactions of these agents with their target cells, this section is mainly focused on approaches involving in vitro research and, when available, we provide a short description of their in vivo application. 

### 3.1. Antibodies as Ligands to Target HER-2^+^ Cancer Cells

Therapeutic antibodies are widely employed in the surface functionalization of targeted nanosystems that can vehicle chemotherapeutic drugs, gene silencing agents or diagnostic molecules for the diagnosis and treatment of HER-2^+^ cancer cells [59]. These antibodies can be anchored to different types of NPs through two main approaches: adsorption or covalent conjugation [57,60]. Functionalization through adsorption methods is simpler, compared to covalent binding strategies, but is less stable. On the other hand, it can facilitate the release of the mAb in the tumor site, enforcing its anti-cancer activity [61]. However, adsorption methods require greater amounts of mAb resulting in a more expensive procedure. Otherwise, covalent binding provides excellent reproducibility and is less subject to spontaneous disassembly. Moreover, covalent binding can be used to obtain orderly antibody orientation when compared with adsorption, since it takes advantage of different conjugation strategies such as carbodiimide chemistry, maleimide chemistry and click chemistry [57,60,62].

TZ is the most widely explored mAb to obtain HER-2 targeted nanosystems for BC therapy [57,61]. In the next paragraph, we focus on the plethora of NPs exploiting TZ conjugation proposed in the last decade. 

Several gold-based NPs, such as nanospheres [63,64,65] and nanorods [66] covalently conjugated with TZ have been proposed to specifically target the HER-2 receptor. In addition, different kinds of polymeric NPs take advantage of the targeting mediated by TZ. Zhang et al. proposed an example of lipid-polymer hybrid NPs electrostatically conjugated with TZ [67], while a similar approach was applied to a novel NP delivery system obtained from polyethylenimine (PEI) and poly (D,L-lactide-co-glycolide) (PLGA) by Yu et al. [68]. Other examples of polymeric NPs exploited a covalent conjugation with TZ to specifically interact with the HER-2 receptor in HER-2^+^ BC [69,70,71], while Dominguez-Ríos et al. proposed a solution based on an HER-2-targeted PLGA nanoplatform covalently biofunctionalized with TZ for HER-2^+^ ovarian cancer [72]. Moreover, magnetic [73,74], carbon-based [75,76] and protein-based [77] NPs conjugated with TZ have been studied.

One of the main advantages of functionalizing NPs with TZ is that the mAb is commercially available, it has been widely used at a clinical level, and its activity is widely demonstrated. On the other hand, one of the main disadvantages of such an approach is the high molecular weight of TZ (approximately 148 kDa) and its relatively large size (around 12 nm) that might significantly modify the physicochemical characteristics of the smallest NPs, and hence their biodistribution. TZ could be used with more success to functionalize bigger NPs, such as polymeric ones that are ten times bigger than the mAb. Another limitation is rapid blood clearance due to increased reticuloendothelial system kidnapping of full antibody-targeted NPs mediated by the Fc recognition site expressed by antigen-presenting cells [78]. Finally, due to their high affinity, anti-HER-2 mAbs can bind HER-2 receptors not only on cancer cells, but also on healthy cells. This specific yet non-selective binding to HER-2 receptors on healthy cells can lead to a reduction in drug concentration at the tumor site coupled with undesired toxic effects in off-target cells/organs [79].

### 3.2. Nanobodies and Antibody-Fragments as Ligands to Target HER-2^+^ Cancer Cells

As an alternative to the use of whole mAbs such as TZ, nanobodies and antibody fragments can be used to address HER-2^+^ BC [80,81]. Indeed, these molecules, smaller in size, succeeded in improving targeting and anticancer activity, since they interfere to a limited extent with the physical and chemical features of the NP [59]. In contrast to what is observed with full antibody-targeted NPs, smaller antibody fragments seem to provide enhanced pharmacokinetic profiles and increased tumor tissue penetration [78,80,82]. 

Among small targeting moieties exploitable to address the HER-2 receptor, single-domain antibodies (sdAb), also known as nanobodies, were recently proposed for their interesting properties [81,83]. Nanobodies are small antigen-binding fragments (~15 kDa) derived from heavy-chain only antibodies present in camelids [84]. It has been reported that these types of molecules display low immunogenicity due to their similarity to human immunoglobulin heavy-chain (VH) sequences, and improved penetration into solid tumors due to their smaller size. Moreover, they have increased solubility and stability compared to full-size human/humanized monoclonal antibodies. Nanobodies targeting a wide range of receptors, including HER-2, have been developed. The HER-2-targeted nanobody 11A4 was used to achieve improved and selective uptake of polymeric NPs in HER-2^+^ BC cells SKBR-3 as compared to HER-2^-^ MDA.MB.231 cells [84], while the sdAb C7b conjugated to silica NPs has been used to delivery drugs, imaging and theranostic agents [81]. Indeed, in vitro studies in SKBR-3 demonstrated a significantly higher uptake of the silica-based-C7b NPs compared to HER-2^-^ larynx carcinoma Hep2 cells, and an improved performance in photodynamic treatment in an SKBR-3 xenograft murine model [81]. 

As an alternative to nanobodies, the use of antibody fragments (Fab) has been evaluated as an HER-2 targeting tool. Similar to nanobodies, Fab are reported to be less immunogenic and, due to their reduced size, should be less prone to alter the physicochemical properties of NPs. Fab obtained by papain cleavage of TZ (TmAb) and panitumumab (PmAb) was employed by Houdaihed et al. to obtain targeted polymeric NPs [80]. In another study, silica NPs were conjugated with the anti-HER-2 Fab-6His, finely tuning ligand density and orientation. In these NPs, the modulation of ligand density and orientation were demonstrated to reduce protein corona formation and to affect specific targeting towards SKBR-3 HER-2^+^ BC in vitro, improving NPs targeting efficacy upon increasing the Fab density [85]. The superiority of Fabs compared to full mAbs as NP targeting molecules was clearly demonstrated by Duan et al. in vitro and in vivo. They compared targeting and accumulation of PLGA-PEG NPs loaded with curcumin and functionalized with full TZ or a particular TZ Fab in BT-474 HER-2^+^ against MDA-MB-231 HER-2^−^ BC cells. The authors reported a five-fold increase in NP accumulation into the tumor mass of heterotopic BT-474 tumor-bearing BALB/c mice when functionalized with the Fab as compared to the full antibody [78].

Single-chain variable fragments (scFv) derived from TZ have also been studied as targeting entities. Recently, Shi et al. reported the development of cell-derived exosome NPs genetically engineered to display two different mAb on the surface. Due to the presence of both anti-human CD3 and anti-human HER-2 derived scFv, this nanostrategy is capable of simultaneously targeting T-cell surface CD3 and cancer cell-associated HER-2 receptors, exhibiting enhanced and specific anti-tumor activity, both in vitro and in vivo, compared to SKBR-3, HCC 1954 HER-2^+^ and MDA-MB-468 HER-2^−^ BC cells using an HCC 1954 xenograft tumor model [86].

### 3.3. Peptides, Aptamers and Ankyrins as Ligands to Target HER-2^+^ Cancer Cells

Among targeting molecules, small peptides derived from TZ have been considered another alternative NP functionalization strategy [79]. The anti-HER-2 peptide AHNP is a small exocyclic peptide derived from TZ. It is able to bind to the HER-2 receptor with high affinity (Kd = 150 nM), inhibiting effectively the receptor’s kinase activity [87,88,89]. This peptide was recently exploited as a targeting moiety for different kinds of NPs: a polymeric nanoconstruct bearing HER-2 specific antisense oligonucleotides [88], liposomal NPs [79] and iron oxide NPs [89]. An innovative approach was proposed by Zhang et al. that uses non-toxic transformable peptides able to self-assemble into micelles under aqueous conditions, following interaction with HER-2 on HER-2^+^ BC cells [90]. Another strategy exploited to target HER-2 is represented by the use of aptamers. These consist of single-stranded DNA or RNA with unique tertiary structures that allow them to specifically bind to target molecules [91]. Smaller size, low immunogenicity, good tissue penetration and easy manipulation are prominent features for their use. Moreover, the implementation of aptamers to functionalize NPs holds great promise for targeted drug delivery and cancer therapy [92]. Recently, several aptamer-decorated NPs have been designed to address HER-2^+^ BC. Gold nanoconstructs conjugated with anti-HER-2 aptamers have demonstrated the ability to recognize HER-2 on SKBR-3 cells and then to be internalized via HER-2-mediated endocytosis. Accumulation of these complexes in lysosomes resulted in accelerated degradation of HER-2 and the suppression of cancer cell growth [93]. In addition, pH-responsive mesoporous silica NPs functionalized with anti-HER-2 aptamers [94], and albumin NPs decorated with a new DNA aptamer named HB5, obtained by exponential enrichment technology, have also been proposed [92]. Moreover, DNA-based nanorobots functionalized with anti-HER-a aptamers have been developed. Ma and coworkers designed and explored in vitro the potential of SKBR-3 cells with a tetrahedral framework of nucleic acids decorated with an anti-HER-2 aptamer, and showed that the construct is able to specifically target HER-2^+^ BC in SKBR-3 cells and induce lysosomal degradation of HER-2 [95].

An interesting targeting strategy as an alternative to antibodies, is the use of designed ankyrin repeat proteins (DARPins). Ankyrins are naturally occurring proteins that act as a link between a variety of cell membrane proteins and the spectrin-actin cytoskeleton in a non-immunoglobulin-based approach [96]. DARPins are recombinant engineered binding proteins with a binding affinity comparable to antibodies, but with smaller size, higher stability, and cheaper production methods [97,98]. A DARPins-based drug (Abicipar pegol) is now under clinical evaluation for age-related macular degeneration [99]. In the context of HER-2^+^ BC, several DARPins have been shown to bind to different extracellular HER-2 domains with low nanomolar affinity and extremely high specificity. This was confirmed in both highly overexpressing SKBR-3 cells and mildly overexpressing MCF7 cells [100]. Moreover, DARPins were used to evaluate HER-2 expression in paraffin embedded tissues from cancer patients, showing again high specificity and equivalence with traditional anti HER-2 immunoistochemistry [101]. In vivo experiments with CD1-FOXn1/nu SK-OV-3 bearing mice showed that by PEGylating DAPRins, their biodistribution and a high tumor-blood ratio but were affected by a rapid clearance, while PEGylated DAPRins had much slower clearance that, on one hand, further increased tumor accumulation, but on the other hand decreased the tumor-blood ratio [102]. In this context, DARPins have been used to functionalize different types of NPs, including magnetic and paramagnetic NPs, gold nanorods, and polymeric NPs as novel diagnostic, therapeutic and theranostic agents [103,104,105] 

Finally, an interesting approach exploits NPs obtained from molecularly imprinted polymers that obstruct the HER-2 signaling by blocking the dimerization site of HER-2 receptors and thereby suppressing the growth of SKBR-3 HER-2^+^ BC as compared to MCF-7 HER-2 basal cells. These imprinted NPs target HER-2 via binding to its glycans differently from the other biochemicals and chemicals, which target HER-2 by binding to one of its domains or sub-domains [106].

In summary, among all the different classes of HER-2^+^ BC targeting nanoagents, it is still impossible to identify the winning strategy. The choice should depend on the final intended application and NP of election. It has already been discussed that small NPs could not be functionalized with whole antibodies without the risk of altering their pharmacokinetics and stability profiles. In parallel, if NPs are developed with a therapeutic goal, the targeting agent should retain the same anticancer activity observed for TZ. Having said that, all reported results seem very interesting and promising and a new class of nanoagents able to specifically target HER-2^+^ BC to deliver cytotoxic or contrast agents would allow us to solve two major unmet clinical needs: (i) the insurgence of resistance mechanisms to available TZ-based targeted therapies and (ii) the difficulties encountered in the process of diagnosis. In this context, the difficulties that physicians have in precisely quantifying HER-2 levels in patients still limit the prescription of proper treatments and the prediction of their efficacy [107]. Both these aspects are evaluated in detail in the following paragraphs. 

## 4. Nanotechnological Strategies for the Treatment of HER-2^+^ BC 

### 4.1. Strategies to Deliver Chemotherapeutic Drugs and Other Cytotoxic Agents

In recent years, NPs functionalized with the above-mentioned HER-2 targeting ligands have been exploited to deliver different kinds of therapeutic agents in HER-2^+^ BC. The vast majority of them have been investigated by in vitro assays at the preclinical level.

Among these, we can find several interesting and convincing works, as summarized in Table 1and Table 2. TZ-coated polymer-based NPs loaded with docetaxel showed increased target selectivity, cellular uptake, and cytotoxicity in HER-2^+^ BT474 cells but not in HER-2^−^ control cells [67]. Cisplatin-loaded lipid, and/or polymer-based NPs, displayed higher in vitro efficacy in comparison to non-targeted NPs and free drug only in HER-2^+^ SKOV-3 but not in HER-2^−^ HCC70 cells [71]. Doxorubicin (DOX), widely exploited for therapeutic purposes in HER-2^+^ BC, has been loaded into polymeric NPs functionalized with an anti-HER-2 antibody and displayed higher cytotoxicity toward HER-2^+^ MCF7 cancer cells than both non-targeted DOX-NPs and free DOX, without significantly damaging healthy cells [69]. Moreover, novel pH-sensitive, aptamer-conjugated mesoporous silica NPs loaded with DOX were described by Shen et al. These NPs released the drug in a pH-dependent manner, along with effective uptake and enhanced cytotoxic effects in HER-2^+^ BC SKBR-3 cells [94]. Several HER-2-targeted polymeric NPs have been studied in vitro for the delivery of paclitaxel (PTX), either alone [68] or in combination with everolimus [74]. PTX has also been delivered to HER-2^+^ BC SKBR-3 cells using iron oxide NPs conjugated with an anti-HER-2/neu peptide, or using worm-like nanocrystal micelles functionalized with TZ [70].

Other works describe the in vivo efficacy of HER-2-targeted NPs loaded with chemotherapeutics. Pegylated carbon-based NPs conjugated with TZ and loaded with DOX have been proposed as well. These NPs are stable under physiological pH conditions but, in presence of the lower tumor environment pH and following endocytosis, DOX is released in a controlled manner resulting in high uptake and activity in vitro and in vivo in HER-2^+^ BC (SKBR-3 models) [75]. Finally, pegylated DOX liposomes conjugated with an anti-HER-2 peptide [108] or with an anti-HER-2 affibody [109] demonstrated good potential in the treatment of a TUBO HER-2^+^ BC model in vivo.

HER-2-targeted NPs have been exploited for intratumor delivery of the ribosome-inactivating protein saporin [84]. PLGA NPs decorated with HER-2 nanobody and loaded with saporin selectively induced cell death of SKBR-3 cells in combination with Photochemical Internalization (PCI). This technique uses a photosensitizer and local light exposure to trigger endosomal escape of entrapped nanocarriers [84].

Another drug encapsulated in TZ-functionalized polymeric NPs is the anti-SRC kinase inhibitor Dasatinib. In this case, the nanoformulation improves its in vitro cytotoxicity against BT474 HER-2^+^ BC cell lines [110]. Curcumin has also been nanoformulated and exploited for BC treatment using SKBR-3 human serum albumin NPs decorated with an anti HER-2 aptamer [92].

An albumin nanoformulation for the delivery of Lapatinib has been suggested, too. The ability of being internalized and inducing apoptosis was confirmed in SKBR-3 cells and stronger anti-tumor efficacy compared to lapatinib alone was determined in tumor-bearing mice with no sub-chronic toxicity [111].

### 4.2. Strategies to Vehicle Nucleic Acids or Gene Silencing Molecules

Short interfering RNA (siRNA) molecules are a class of double-stranded non-coding RNA molecules. In recent years, they have been extensively studied in BC as an emerging class of drug, since they are able to regulate gene expression. With the use of siRNAs, it is possible to inhibit specific genes and perform cancer treatment of undruggable targets. However, siRNAs cannot not be employed in vivo as such, since they are affected by inherent instability [112]. In the attempt to overcome issues of resistance to conventional therapies, Gu et al. developed mesoporous silica NPs conjugated with HER-2-targeting antibodies to deliver HER-2 siRNA, demonstrating in vitro that HER-2^+^ BC cells usually do not develop resistance to HER-2 siRNA compared to TZ or lapatinib. Moreover, this siRNA can silence HER-2 at the mRNA level, preventing adaptational changes and survival mechanisms in BT474, SKBR-3 and HCC1954 cells. The authors conclude that ablation of HER-2 receptor using HER-2 siRNA [113] could overcome the reactivation of signaling in a BT474 in vivo model, typical of cells resistant to different chemotherapies (e.g., lapatinib) [114,115].

Cristofolini at al. studied a magnetic hybrid nanostructure of iron oxide stabilized by caffeic acid coating for magnetic responsive delivery of negatively charged siRNA entrapped in a middle layer of calcium phosphate. This structure, externally coated with PEG to improve colloidal stability and evading immune system recognition, is able to enhance the delivery of HER-2 siRNAs to a human HCC1954 BC cell line in vitro [116]. In another work, siRNA delivery was improved by assembling RNA-based NPs designed to resist nucleases. This novel nanostructure has highly efficient gene silencing ability, tumor targeting specificity, and chemical and thermal stability. In addition, a 2′-F-modified 3WJ-HER-2aptasiXBPI siRNA has been incorporated in the NPs, becoming more metabolically and structurally stable and displaying a higher renal filtration clearance [117]. 

Another advantage of using NPs is the possibility of co-loading them with more than one agent to perform a dual therapy. Indeed, the combination of a newly developed HER-3 siRNA and TZ has been used for the treatment of HER-2^+^ BC using polyethyleneimine-functionalized carbon dots. Overexpression of HER-3, a member of ErbB receptor family, is associated with TZ resistance. In recent studies, it has been demonstrated that siRNAs that targets HER-3 can increase antitumoral efficacy of TZ in HER-2^+^ cancer cells. Therefore, a combination therapy of TZ and an HER-3 specific siRNA is effective in blocking HER-2/HER-3 signals, since increased tumor penetration downregulates HER-3 overexpression and is able to inhibit proliferation of BT474 cells without inducing resistance to the therapy [76].

Another study reported the use of a polymalic acid-based mini nanodrug attached to HER-2-specific antisense oligonucleotides and peptides able to target HER-2 receptors on BT474 cells [88]. Here, the authors claim that the shape and size of their mini nanodrugs are important in enhancing penetration of multiple bio-barriers by the nanoconstructs to enhance their therapeutic efficacy.

Finally, NPs have been used to deliver the mRNA of TZ in vivo to overcome many of the gaps in antibody production and therapeutic application. The mRNA encoding for TZ was protected from degradation by encapsulation into lipid-based NPs designed to allow liver-targeted production of TZ. In this way, prolonged expression of full-size TZ was achieved in the liver, providing an effective cancer treatment and offering a valuable alternative to protein administration [118]. 

### 4.3. Nanostrategies to Improve Radiation Efficacy in HER-2^+^ Tumors

Radiation therapy is widely used both in the treatment of early and advanced stage BC and as palliative treatment to mitigate pain in patients with metastatic BC. This procedure takes advantage of high energy beams to kill cancer cells and shrink tumor masses. 

Gold pegylated NPs linked to TZ and complexed with Auger electron-emitter 111In, showed inhibition of tumor growth in vitro and in vivo (SKBR-3 models) without toxicity to normal tissues. In addition, the author showed the critical role of functionalization in increasing the internalization capacity of NPs in HER-2^+^ cells [64]. In another report, TZ-modified AuNPs labeled with ^177^Lu were developed to target the HER-2 receptor on SKBR-3 and BT474 BC cells, and were more effective in inhibiting tumor growth in vivo compared with non-targeted AuNP-^177^Lu [119]. Magnetite NPs can be exploited in antitumor local radiation. Modifying the magnetite core using α emitter ^225^Ac, it is possible to obtain a combination of ionizing radiation and magnetic hyperthermia in a single drug, while the TZ conjugation confers tumor targeting specificity in SKOV-3 cells [73].

A completely new radiation therapy, named α-nanobrachytherapy, has been proposed for solid unresectable tumors. This approach has been utilized for selective therapy of HER-2^+^ BC and involves the injection of 5 nm diameter gold NPs labeled with an ^211^At emitter, and the α irradiation of bismuth target. NPs used in this approach are stabilized with PEG and conjugated with TZ to achieve target selectivity. ^211^At-AuNPs-PEG-TZ can be effectively used as a local therapy for HER-2^+^ cancers, as suggested by in vitro studies with ovarian SKOV-3 cancer cells [65]. 

Radioactive upconversion NPs (UPNPs) coupled with the beta-emitting radionuclide yttrium-90 (90Y) and functionalized with specific DARPins binding HER-2 (DARPIn 9-29) were shown to improve therapeutic efficacy in SK-BR-3 HER-2^+^ cells compared to HER-2^−^ CHO cells. Moreover, these NPs confirmed their therapeutic activity in HER-2 xenograft tumors in vivo, where they delayed and reduced tumor growth after intratumor injection [120]. Even if promising, this approach should be further improved by carefully evaluating off-target toxicity after systemic administration. 

Another emerging field of local radiation is represented by photothermal therapy (PTT). PTT is a minimally-invasive therapeutic approach based on the toxic effect produced when specific photothermal agents that accumulate into the tumor are locally irradiated with an external source to convert this energy into heat that shrinks the tumor mass. NPs with the ability of specific tumor accumulation and high photothermal conversion rate have been proposed to improve PTT efficacy. Among these, gold nanorods display interesting properties. Indeed, by tailoring the size and shape of nanorods it is possible to tune the near infrared wavelength of absorption, transforming them into extremely good photothermal agents [66]. However, bare gold nanorods cannot effectively target the tumor, thus limiting their efficacy. Therefore, Kang et al. designed gold nanorods (GNRs) functionalized with TZ and porphyrin, obtaining improved targeting specificity and significantly higher inhibition of tumor growth in a BT474 xenograft in vivo model [66]. This study showed how GNRs represent a promising tool for the treatment of HER-2^+^ BC. DARPins have also been used as targeting moieties to improve SKBR-3 HER-2^+^ cell accumulation of UCNPs to be used as PTT agents. This approach was confirmed in vivo in a Lewis lung cancer (LLC) mouse model, in which the administration of functionalized UPNCs reduced tumor growth after a single laser irradiation [121]. 

**Table 1:** Summary of all significant examples of organic NPs developed for HER-2^+^ BC therapy.

Mechanism of Action and *Targeting Molecule*		Achievements	NP Type		Reference
**Antibodies**	Targeted delivery of anticancer drug. *TZ (LuyePharma, Yantai, China/Genentech, South San Francisco, CA, USA)*	- Increment of anticancer activity, decrease of toxicity towards healthy cells - Lower chemotherapeutic dose required for treatment	Polymeric/lipid	in vitro	[67,68,69,70,71]
	Delivery of in vitro-transcribed mRNA coding for TZ	- Improved PK profile - Suppression of tumor growth - Reduction of off-target effects	Lipid	in vitro—in vivo	[118]
	Targeted delivery of anticancer drug. *TZ (Roche, Basel, Switzerland)*	- Prevention of chemoresistance - Higher cytotoxicity on cancer cells	Polymeric	in vitro	[72]
	Release of anticancer drug in combination with *TZ (Roche, Basel, Switzerland)*	- Effective neoadjuvant therapy with reduced toxicity	Albumin-bound Paclitaxel (nab-PTX)	clinical trial	[77]
	Targeted delivery of anticancer drug. *TZ (Roche, Basel, Switzerland)*	- Improved NP stability - Induction of apoptosis and cell cycle arrest	Polymeric	in vitro	[110]
**Nanobodies and antibodies fragments (Fab)**	Dual-targeted delivery of chemotherapeutics to HER-2 and EGFR. *TZ—Panitumumab Fab fragments (In-house recombinant production)*	- Enhanced tumor accumulation - Higher cellular internalization and cytotoxicity	Polymeric	in vitro	[80]
	Targeted delivery of cytotoxic molecule after photochemical internalization. *11A4-nanobody (In-house recombinant production)*	- Selective uptake - Strong inhibition of cell proliferation	Polymeric	in vitro	[84]
	Targeted delivery of cytotoxic agent. *TZ fragment (Genentech, South San Francisco, CA, USA)*	- Enhanced cellular accumulation and higher cytotoxicity - Higher tumor permeability and in vivo half-life	Polymeric	in vitro—in vivo	[78]
	Induction of tumor specific immune response. *anti-HER2-anti-CD3 dual-scFv*	- Enhanced stability - Controlled antitumor immunity - Minimal toxicity and immunogenicity	Cell-derived exosome	in vitro—in vivo	[86]
**Peptides**	Delivery of antisense oligonucleotide. *NH2-PEG200-AHNP*	- Prevention of HER2 receptor synthesis - Inhibition of cancer cell proliferation - Reduction of tumor growth	Polymeric	in vitro—in vivo	[88]
	Targeted delivery of cytotoxic agent. *HER2pep YCDGFYACY-MDV (In-house recombinant production)*	- Higher binding activity to cancer cells and endocytosis of the drug - in vivo anti-tumor efficacy with minimal toxicity	Liposome	in vitro—in vivo	[79]
	Generation of nanofibers able to disrupt HER2 dimerization. *HER2pep BP-FFVLK-* *YCDGFYACYMDV*	- Induction of cancer cell apoptosis - Effective in mouse xenograft model	Peptide-based	in vitro—in vivo	[90]
	Targeted delivery of anticancer drug. *AHNP* *(FCDGFYACYADVGGG)*	- Higher uptake in tumor cells - Increased cytotoxicity and prevention of tumor growth	Liposome	in vitro—in vivo	[108]
**Aptamers**	Targeted delivery of anticancer molecule. *HB5 DNA aptamer*	- Good size distribution, solubility and long erm stability - Higher cytotoxic effect	Albumin-based	in vitro	[92]
	XBP1 deletion by therapeutic siRNA delivery. *3WJ-HER2 aptamer (In-house recombinant production)*	- Higher tumor cell targeting and lower binding to heathy tissues - Increased gene silencing, suppression of tumor growth and prevention of drug resistance	RNA-based	in vitro—in vivo	[117]
	Lysosomal degradation of membrane protein HER-2. *HApt aptamer (TaKaRa Ostu, Japan)*	- Induction of cell apoptosis and inhibition of cell proliferation - Enhanced stability and circulation time	DNA nanorobot	in vitro—in vivo	[95]

**Table 2:** Summary of all significant examples of inorganic NPs developed for HER-2^+^ BC therapy.

Mechanism of Action and *Targeting Molecule*		Achievements	NP Type		Reference
**Antibodies**	Intratumor retention leading to an immune response activation. *TZ (Genentech, South San Francisco, CA, USA)*	- Antitumor immune response without requiring a therapeutic payload - Reduced tumor growth	Iron oxide	in vitro—in vivo	[74]
	Targeted photothermal ablation by near-infrared laser. *TZ (Roche, Basel, Switzerland)*	- Enhanced targeting specificity - Inhibition of tumor growth - Limited damaging to surrounding tissues	Gold nanorods	in vitro—in vivo	[66]
	Selective targeting of HER-2 Increase of pro-apoptotic proteins. *TZ (Roche, Basel, Switzerland)*	- Higher stability and cellular internalization - Higher cytotoxicity related to survival-proliferation pathways decrease	Gold nanospheres	in vitro	[63]
	HER-2 gene silencing by siRNA delivery. *TZ (Roche, Basel, Switzerland)*	- Reduced proliferation and prevention of tumor initiating cells - More durable inhibition than existing therapeutic monoclonal antibodies and small molecules	Carbon dots/mesoporous silica	in vitro	[76,114]
	Radio-immunotherapy Magnetic hyperthermia. *TZ (Roche, Basel, Switzerland)*	- Enhanced cytotoxicity due to internal irradiation - Suitable for destroying micrometastatic cancer cells thanks to the reduced size	Superparamagnetic iron oxide	in vitro—in vivo	[73]
	Antitumor local radiation. *TZ (Roche, Basel, Switzerland)*	- Specific tumor cell binding and internalization and higher cytotoxicity - Inhibition of tumor growth - Suitable for the elimination of single micrometastatic cancer cells	Gold NPs	in vitro—in vivo	[64,65,119]
	Targeted delivery of anticancer molecule. *TZ (Roche, Basel, Switzerland)*	- Higher cellular uptake and lower toxicity - Efficient inhibition of antitumor activity	Carbon-based	in vitro—in vivo	[75]
**Nanobodies and antibodies fragments (Fab)**	Local irradiation (PDT). *sdAb C7b* *(In-house recombinant production)*	- Higher uptake rate - Local hyperaemia, oedema, necrosis after the first irradiation - Modest effect of PDT	Silica	in vitro—in vivo	[81]
	Improved targeting due to the reduced protein corona formation. *Anti-HER2 Fab-6His (Hangzhou HealSun Biopharm Co., Ltd., Zhejiang, China)*	- Enhanced cytotoxic effect	Silica	in vitro	[85]
**Peptides**	Targeted delivery of anticancer molecule Inhibition of kinase activity. *AHNP* (GenScript Inc., Piscataway, NJ, USA)	- Great stability in biological medium thanks to size and uniform shape - Improved targeted delivery of drug in vivo and in vitro	Iron oxide	in vitro—in vivo	[89]
**Aptamers**	Targeted delivery of anticancer molecule Downregulation of HER-2 Hapt is also an antagonist Synergic mechanism. *HApt aptamer (Sangon Biotech Co., Ltd., Shanghai, China)*	- Inhibition of cell proliferation by induction of apoptosis	Mesoporous silica nanocarrier	in vitro	[94]
	Downregulation of HER-2. *(HApt aptamer IDA Inc., Coralville, IA, USA)*	- Efficient lysosomal targeting - Inhibition of cell proliferation - Suitable for PTT applications	Gold nanostars	in vivo	[93]
**DARPins**	Targeted delivery of 90Y radionuclides and toxins. *DARPin_9.29 (in-house recombinant production)*	- Specific cytotoxicity of DARPin-^90^Y-UPNPs in SKBR-3 cells - Reduced tumor growth after ^90^Y-UPNPs intratumoral injection in vivo	Upcoversion NPs	in vitro—in vivo	[120]
	Photothermal therapy. *DARPin_9.29 (in-house recombinant production)*	- Specific cytotoxicity in SKBR-3 cells after laser irradiation - Reduced tumor growth after laser irradiation in vivo	Upcoversion NPs	in vitro—in vivo	[121]

## 5. Detection of HER-2^+^ BC: State of the Art and Clinical Needs

As already mentioned, HER-2 is an important clinical biomarker, as its overexpression or amplification is associated with greater aggressiveness and influences a patient’s management [122,123]. Therefore, a precise quantification of HER-2 levels in patients is of fundamental relevance to guide doctors in elaborating the proper therapeutic strategy and predicting the response to therapy. The standard methods used in clinics to define HER-2 positivity are: (i) IHC, and (ii) FISH and chromogenic in-situ hybridization (CISH). IHC shows the amount of HER-2 protein in the sample, while FISH allows the determination of the number of copies of HER-2 present in tumor cells [124]. CISH is a diagnostic method that combines the chromogenic signal detection method of IHC techniques with in situ hybridization [125].

Despite their wide use, these techniques have several limitations. First of all, IHC, FISH and CISH are highly invasive since they require tissue biopsies. Furthermore, their sensitivity and target specificity still need to be improved [125]. It has been reported that about 20% of HER-2 positivity diagnoses made with the available standard methods appear to be inaccurate [126,127]. Moreover, HER-2 expression in primary tumors and metastases is highly discordant, with several reported cases of HER-2^−^ primary and HER-2^+^ metastases. Therefore, several biopsies should be made during the course of treatment, at least in uncertain cases, and not all metastatic foci are available for sampling. In addition, HER-2 gene overexpression may not correlated with protein levels, and FISH results may underestimate HER-2 levels. These aspects may significantly contribute to imprecise quantifications obtained by standard methods [128]. Based on these considerations, it appears clear that the development of tools able to target HER-2 with high sensitivity, and in a simple and accessible way, would be of great help to enable an early diagnosis of the disease, thus guiding therapeutic choice and increasing the chances of survival [58]. These targeting agents should be able to evaluate HER-2 levels in primary tumors and small metastases, as well as working on circulating tumor cells to enable liquid biopsies [129].

## 6. Nanotechnological Approaches for HER-2^+^ BC Diagnosis

**Figure 2:**
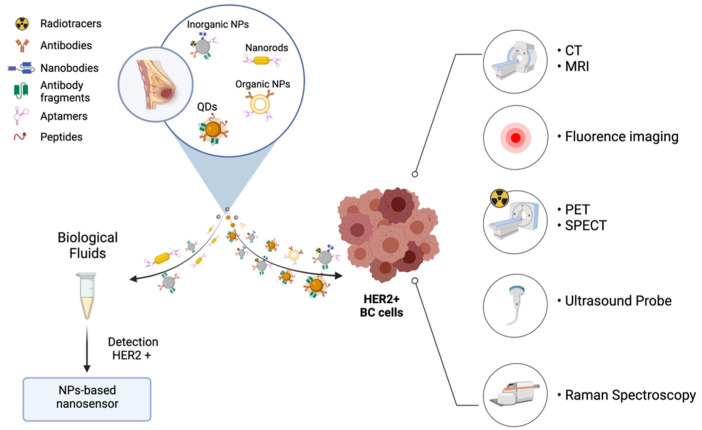
Nanotechnology approaches for HER-2^+^ BC diagnosis.

Recently, several nano-based approaches have emerged as promising candidates for the detection and screening of HER-2^+^ BC, including organic and inorganic NPs, QDs and aptamers (Table 3 and Figure 2) [58].

### 6.1. Imaging Agents for Early Diagnosis and Tailored Therapy

Using innovative bioimaging techniques, HER-2-targeted NPs can allow oncologists to evaluate HER-2 expression more precisely than with traditional methods and to elaborate the correct therapeutic strategy more rapidly. The imaging techniques that have shown the most promising results are computerized tomography (CT), positron emission tomography (PET), single photon emission computed tomography (SPECT), ultrasounds (US), magnetic resonance imaging (MRI) and optical imaging [58,130,131]. These techniques have their own advantages and disadvantages. PET and SPECT have a much higher sensitivity compared to MRI, but much lower resolution and cannot provide any anatomical information [132].

Most of the targeting agents exploit the anti-HER-2 humanized monoclonal antibodies (Trastuzumab and Pertuzumab), mAbs, Fabs, scFv, etc. Labeling NPs with these targeting moieties has the dual advantage of conferring stability to the targeting agent and loading a single vector with many contrast agents, thus significantly increasing the specificity and intensity of the signal [133]. Moreover, the possibility of modifying the physico-chemical properties of the constructs, allows a much higher control of the biodistribution and clearance kinetics, thus improving tumor retention and allowing prolonged imaging of the tumor tissue [134].

#### 6.1.1. Magnetic Resonance Imaging and Computerized Tomography

MRI is a widely diffused non-invasive imaging tool used in the clinics for diagnosis of tumors. It is characterized by a high degree of soft tissue contrast, and spatial resolution without any limitation due to signal depth. To increase contrast, exogenous contrast agents are administered prior to scanning [132]. Chen et al., explored the possibilities of using superparamagnetic iron oxide NPs modified with dextran and conjugated with Herceptin for detecting HER2 in vitro and in vivo. The authors reported significant magnetic resonance enhancements for the different BC cell lines tested proportional to the HER2/ neu expression level (SKBR-3, BT-474, MDA-MB-231, and MCF-7). When TZ–NPs were administered in vivo, the authors observed an accumulation of the contrast agent in the tumor sites, confirming their suitability for detection of HER2/neu-expressing BC [135].

In another work, PEGylated SPIONs were exploited to target HER-2^+^ BC. The authors functionalized the particles with a single chain variable fragment directed against HER-2 and labelled them with a Cyanine (Cy) based fluorescent tag to provide dual tracking modality. These NPs demonstrated enhanced uptake into BC cells depending on their HER-2 expression level (BT474, SKBR-3, MDA-MB-231 and MCF-7) [136]. These results, together with their biodegradable nature and biocompatibility profile, represent prominent features for their use in early BC diagnosis.

Another approach is so called “supersensitive magnetic resonance imaging”, that uses contrast agents with a high R2 relaxivity (low T2, as R2 = 1/T2) to enhance imaging sensitivity. In this context, Zhang et al., developed recombinant magnetosomes functionalized with an anti-HER-2 affibody that showed high specificity only for the SKBR-3 HER-2^+^ BC model up to 24h after IV administration. Interestingly, the immunogenicity of the complex was controlled by chemically removing the lipid bilayer of the extracted magnetosome with a non-pyrogenic stealth lipid mixture. Moreover, the highly negative zeta-potential of the system (≈−20 mV) significantly reduced the nonspecific binding of magnetosomes to non-target cells. These small details confer to the study an even stronger translation potential toward safe clinical applications [137].

#### 6.1.2. In Vivo Fluorescence Imaging

Fluorescence based techniques are widely used at a clinical level for all sorts of diagnostic assays. NIR infrared fluorescence bioimaging is emerging as a powerful technology especially for fluorescence-guided surgery, with many dyes already used at a clinical level, such as indocyanine green. Many tools are being tested preclinically, such as organic natural fluorescent dyes, hybrid fluorescent dyes, protein dyes, polymer dots, and quantum dots. 

Quantum dots (QDs) are characterized by a tunable and narrow fluorescence emission spectrum, long lifetime and high optical stability compared to other organic fluorescent dyes [138]. However, their clinical development is limited by toxicity issues, as they are made using heavy metals [139]. Wei et al. developed a method for the identification of HER-2^+^ BC using an HER-2 antibody with MnCuInS/ZnS QDs-loaded BSA fluorescence NPs [140]. Interestingly, the authors developed a method to produce heavy metal-free QDs, thus addressing the main concerns related to QD toxicity. In this work, the nanoprobe was tested in HER-2^+^ SKBR-3 cells versus HER-2^−^ MDA-MB-231 cells. After 4 h of incubation, the authors demonstrated by confocal microscopy that functionalized NPs were able to specifically target HER-2^+^ cells with very low cytotoxicity. In another in vitro study, the direct conjugation of the anti-HER-2 antibody to the QD surface for detection of the HER-2 receptor was investigated in HER-2^+^, and HER-2^−^ cells. An increase in the uptake of anti-HER-2-QD antibody conjugate was found only in SKBR-3 HER-2^+^ cells [141]. Wang and colleagues screened two novel HER-2 targeting peptides, YLFFVFER and KLRLEWNR, and conjugated them to quantum dots to specifically target HER-2^+^ in vivo in an SKBR-3 model [142]. Another study reported the possibility of using QDs conjugated with HER-2 targeting single-domain antibodies (sdAb), for detecting micrometastases and disseminated HER-2^+^ tumor cells ex vivo [143].

Further, in vivo biodistribution and imaging studies will be important to confirm if these promising results can be translated in clinics, carefully evaluating targeting specificity and sensitivity, to detect single cells ex vivo.

#### 6.1.3. Nuclear Medicine

Nuclear imaging techniques such as PET and SPECT represent powerful tools to allow HER-2^+^ BC visualization. These have been the first clinically approved molecular imaging modalities and nowadays are widely diffused in clinical practice, with ^18^F-fluorodeoxygluocse and ^99m^Tc being the most common tracers used for PET and SPECT respectively [130]. However, these agents lack any tumor specificity, and researchers have been developing several engineered tracers with cancer targeting ability. In the case of HER-2^+^ cancers, numerous studies have developed radiotracers conjugated with TZ, TZ antibody fragments, affibodies, nanobodies and aptamers to allow specific tumor targeting. Among these, Xavier C. et al. used ^18^F labelled with an anti HER-2 nanobody as a PET contrast agent and demonstrated a significantly higher tumor-to-blood ratio, at 1h and 3h after injection, for the targeting tracer compared with non-targeting tracer in a SKOV-3 xenograft model. All agents were quickly cleared through the kidneys [144]. Similarly, Ahlgren et al. used anti HER-2 affibodies labelled with ^99m^Tc as specific SPECT cancer imaging agents in two in vivo BC xenograft models with different HER-2 expression. Interestingly, they showed high tumor accumulation of the engineered probes 4h after injection, and this was proportional to the HER-2 expression levels. Moreover, with a modification to the C-terminal cysteine of the construct, they were able to reduce off-target liver uptake [145]. 

Although interesting, these solutions have limitations, such as low contrast and short half-life, that limit the temporal window to obtain optimal imaging acquisitions. Due to advantages offered by nanotechnology, such as high loading capacity and multiple surface functionalization, it has been possible to develop radiolabeled probes with higher contrast and favorable biodistribution kinetics compared to standard imaging probes [146]. However, most of the studies found in the literature rely on passive tumor targeting, mediated by the so-called Enhanced Permeation and Retention (EPR) effect [147], while only a few works exploit specific surface functionalization to enhance tumor targeting. An example of specific tumor targeting in an HER-2^+^ BC attempt was described by Rainone et al. [148]. The authors developed ^99m^Tc-radiolabeled silica NPs functionalized with a TZ half-chain and verified the nanoprobe specific affinity with SKBR-3 HER-2^+^ BC cells in vitro, and in the relative xenograft animal model ex vivo. 

Tumor radioactivity was higher in animals treated with TZ -functionalized probes as compared to non-functionalized ones. However, this difference was not statistically significant since at longer time points the advantage of functionalization was lost [148]. 

These results suggest that further research needs to be done to optimize NP characteristics and imaging set up to confirm the potential impact of nanotechnology in promoting HER-2 targeting probes for nuclear imaging. Other studies have co-loaded nuclear medicine radiolabels and chemotherapeutic agents inside the same nanocarriers to combine diagnosis and therapy in the so-called theranostic [149,150]. These are addressed in a dedicated section at the end of this review (Section 7).

#### 6.1.4. Multimodal Hybrid Approaches

As previously said, all imaging techniques have intrinsic advantages and disadvantages. A possible solution would be coupling more than one imaging approach within the same nanoplatform. This is a very powerful approach that could lead to improved diagnosis and should be explored in detail. 

An early work using multimodal imaging was reported by Jang et al. [151]. The authors used silica core shell NPs, both fluorescently and magnetically labelled, for fluorescence imaging and MRI (in vitro). The particles were targeted with TZ and provided high contrast in HER-2^+^ SKBR-3 cells. Even if very preliminary, these NPs could be interesting agents for further multimodal in vivo imaging applications. Similarly, Li et al. used DARPin G3-coated fluorescently labelled SPION for HER-2^+^ cells and a tumor model in vitro and in in vivo, respectively. The authors showed that functionalized SPIONs had a significantly higher binding with SKBR-3 HER-2^+^ cells than with MDA-MB-231 HER-2^−^ ones. This behaviour was also observed in mouse models, in which, by MRI, much higher contrast in SKBR-3 tumor bearing mice than in MDA-MB-231 bearing mice was observed [104]. Interestingly, the DARPin G3 has a different binding site compared to TZ, suggesting that DARPin G3 based HER-2 targeting ability should not be influenced by treatment with TZ.

Ultrasound molecular imaging has undergone significant evolution due to the development of specific ultrasound contrast agents offering high tumor selectivity and high sensitivity [152]. Li et al. used US and MRI in a SKBR-3 in an in vivo model to evaluate the targeting ability and biodistribution of iron oxide-doped silica NPs functionalized with an anti-HER-2 antibody. Even if the synthesized NPs had a short blood half-life (with a peak 60 min after administration) and high liver uptake, mainly due to their large size (diameter of approximately 200 nm), the authors observed specific intratumoral accumulation of NPs both by US and MRI within minutes after administration [153]. Even if the increase in image contrast was not significant between the target and the control group, and the possibility of visualizing metastases was not explored, the idea of coupling these two imaging modalities is extremely interesting. 

In another work by Chen et al., MRI was coupled with CT, demonstrating the possibility of specifically imaging an HER-2^+^ in vivo model with a generation 5 Polyamidoamine (PAMAM) dendrimer covalently linked to gold NPs and gadolinium, and functionalized with TZ [154]. The authors reported a significant increase in both MRI and CT contrast (30% and 13% respectively) 4 h after IV administration of functionalized dendrimers, as compared to non-functionalized ones. Biodistribution and MRI proved the persistence of functionalized dendrimers in the tumor up to 48h, while the signal in non-target organs started decreasing 4h after administration. The specificity and the possibility of such a wide imaging window are the major strength of this nanotool, that may allow early and non-invasive diagnosis of both HER-2^+^ primary tumors and metastases. 

Other approaches to address HER-2^+^ BC, involve the use of SPIONs-Cy-PEG-scFv as a targeted imaging agent in vivo. The authors reported a significant increase of MR signals in BT474 HER-2^+^ tumor-bearing mice 24h after injection, compared to non-functionalized NPs, indicating specificity scFv versus HER-2 overexpressing cells/tumors [136].

In 2018, Chen et al. showed how dual radio (89Zr)-labelled and fluorescently (Cy5.5)-labelled ultra-small silica NPs functionalized with anti-HER-2 scFv fragments specifically accumulated in HER-2^+^ BT474 tumors [155]. Perfectly aware of the off-target distribution issue, the authors explored NPs with proper size and surface characteristics to avoid off-target distribution in the liver and limited renal filtration. After 24h, the particles seemed to reach a peak concentration in the tumor that was maintained at an approximately stable level until 72 h after administration. By contrast, the off-target signal slightly decreased. Results showed the highly specific tumor accumulation and clear targeting potential of such NPs. 

### 6.2. Nano-Biosensors for the Detection of HER-2 Levels in Biological Fluids and Tissues

Another huge area of research is the development of novel nano-biosensors able to detect and quantify HER-2 in blood or small tissue samples collected from patients. This approach is of extreme interest and would allow an early diagnosis of HER-2^+^ BC, the possibility of predicting recurrence, detecting metastases, and improving treatment regimen in the case of low response and acquired resistance to target therapies [156]. 

#### 6.2.1. Inorganic NPs-Based Nanosensors

A successful nanotechnological non-invasive approach for detecting and quantifying HER-2 levels in serum samples obtained from patients was developed by Emami et al. [157]. The authors developed a label-free immunosensor consisting of pegylated iron oxide NPs conjugated with anti-HER-2 antibodies. This non-invasive strategy was highly sensitive, responding to HER-2 concentrations over the ranges of 0.01–10 ng mL^−1^ and 10–100 ng mL^−1^. In addition, the simplicity and accuracy of this method make it comparable to other methods responsive to HER-2. 

Apart from the more diffused Iron Oxide Nanoparticles (IONPs), magnetite NPs have been explored as magnetic nano-biosensors. In a preliminary report, Villegas-Serralta et al. developed aminosilane-coated and dextran-coated magnetite nanoparticles (As-M and Dx-M, respectively) conjugated with anti-HER-2 scFvs and evaluated their potential use as biosensors for HER-2 detection using a magnetic based ELISA [158]. The authors showed that As-M NPs were more efficient in scFv immobilization and had higher biomarker targeting activity. An advantage of such an approach is that As-M were also detectable by Raman-Spectroscopy, which is emerging as a very promising technique for HER-2 detection and quantification. Yang et al., developed a novel surface-enhanced Raman scattering (SERS) probe to distinguish between HER-2^+^ SKBR-3 cells and HER-2^−^ MCF7 cells [159]. Gold NPs have also been employed as HER-2 nanosensors. Tao et al. developed a nanoplatform based on gold nanoclusters encapsulated into liposomes further functionalized with an anti-HER-2 antibody or aptamer [160]. The authors proved that their cost-effective nano-biosensor are highly biocompatible and can precisely quantify HER-2 levels in cell suspensions and identify HER-2^+^ cancer cells on tissue slices. As compared to traditional immunoassays, the main advantages of such an approach are related to the intrinsic robustness of gold NPs and to the significantly higher sensitivity due to the high loading capacity of gold NPS inside a single liposome. 

Finally, examples of QDs-based HER-2 nanosensors can be found in the work by Tabatabaei-Panah [161]. Herein, they provided the proof of concept of anti HER-2 functionalized QDs as sensors to specifically bind HER-2 over-expressing SKBR-3 cells and tumor tissues ex vivo. The authors used a homemade anti HER-2 antibody-biotin conjugate labelled with commercially available QDs (QD525, Invitrogen) in comparison with FITC-labelled antibodies. In both in vitro and ex-vivo experiments, immunofluorescence analysis showed that labelling with QDs improved the staining index (contrast between specific HER-2 signal and background) by five times compared with FITC. These results are remarkable, and the images in the paper are impressive. However, the authors fail to provide sufficient evidence for the specific labelling of HER-2, since preliminary results were not supported by further histopathological analyses.

#### 6.2.2. Aptamers Based Nanosensors

Another approach for the detection of HER-2^+^ cancer cells in fluids and tissues is based on the use of aptamer-based organic NPs. Gijs et al. discovered two DNA aptamers (HeA2_1 and HeA2_3), by screening a library of ligands obtained by a whole-cell systematic evolution and exponential enrichment (SELEX) method to bind HER-2 protein with high specificity. Their results demonstrated that both aptamers possessed excellent binding affinity to HER-2^+^ cell lines (SKOV-3 and SKBR-3) and an HER-2^+^ tumor tissue sample in comparison to an MDA-MB-231 cell line (low HER-2 expression level) [162]. Chu et al., developed another HER-2 aptamer (HB5) using the SELEX approach. The HER-2 HB5 aptamer was able to specifically bind to HER-2^+^ BC cells (SKBR-3) with minimal binding to HER-2^−^ cells (MDA-MB-231). Furthermore, an improved IHC method with an HB5 aptamer was investigated in clinical samples compared to other commercial kits approved by the FDA for the evaluation of the HER-2 expression profile, demonstrating greater efficacy and specificity than commercial kits [163].

Aptamers have also been developed by Kim et al. and used as plasmonic nanosensors for HER-2 in biological fluids. In a preliminary study, the authors coupled anti-HER-2 specific aptamers to dopamine-coated gold nanorods and verified an extremely low limit of detection, thus opening the way to super-sensitive detection methods [164].

## 7. Theranostics

The is great interest in multifunctional NPs that can be used as theranostic agents able to: (i) specifically visualize primary tumors and metastases; (ii) to quantify HER-2 levels allowing more accurate tumor stratification, and (iii) specifically deliver therapeutic agents to HER-2^+^ tumors. 

In this context, Zheng et al. reported the use of multivalent PLGA NPs loaded with SPIONs and DOX, further labelled with gold NPs and Herceptin. These were used as MRI and photoacoustic dual contrast agents to couple the advantages of both imaging techniques. Furthermore, they promoted both DOX-driven chemotherapy and photothermal therapy due to the presence of gold NPs on the surface of PLGA NPs. These NPs resulted in a unique and complex nanosystem able to modulate immune responses and remodel the tumor microenvironment in a BT474 xenograft model amplifying the antitumor therapeutic effect [165]. 

Rainone et al. used fluorescent and ^99^mTc-radiolabeled silica NPs functionalized with a TZ half-chain to specifically detect HER-2 overexpressing tumors. Moreover, they loaded the NPs with DOX to simultaneously confer traceability and antitumoral properties in comparison with liposomal DOX (Caelyx) [150]. The developed nanoagents could be utilized as new theranostic agents for HER-2^+^ BC lesions. The authors showed high accumulation of NPs in the SKBR-3 tumor mass within the first 4h of treatment, with a fast decay and low tumoral specificity at later time points. This peculiar aspect should be improved to grant longer tumor visualization and to allow the use of these NPs during surgical procedures.

Choi et al. developed pluronic-based NPs functionalized with an anti-HER-2 antibody and labeled with Cy5.5 to enable in vivo optical imaging. The authors co-loaded IONPs and DOX to simultaneously couple MRI and chemotherapy. Specific cellular uptake was studied in HER-2 overexpressing SKBR-3 cells. Moreover, in an in vivo xenograft tumor, the developed herceptin-functionalized NPs showed higher tumor uptake and antitumor efficacy compared to non-functionalized ones 14 days after treatment [166]. 

All these examples can be further improved in terms of cancer specificity, off target distribution, and metastases detection to enhance their success in clinical translation. As a general consideration, coupling early detection and therapy could provide an enormous advantage toward future clinical applications to reduce intervention invasiveness and improve treatment schedule.

**Table 3:** Summary of all significant examples of NPs developed for HER-2^+^ BC diagnosis.

Technique	NP Type	Achievements		Reference
**Magnetic Resonance Imaging**	*Herceptin*-dextran iron oxide nanoparticles *(Roche, Basel, Switzerland)*	- Low cytotoxicity - Magnetic resonance enhancements proportionally to the HER-2/neu expression level in vitro - Higher level of accumulation of the contrast agent in tumors expressed the HER-2/neu receptor	in vitro—in vivo	[135]
	SPIONs-Cy-PEG-scFv *(Recombinant scFv 4D5-Cys)*	- Higher affinity and specificity in vitro - Selective MRI labelling of HER-2^+^ tumors in vivo	in vitro—in vivo	[136]
	Magnetosomes functionalized with an anti-HER-2 affibody *(Recombinant MamC (GenBank: CDK99608.1) and anti-HER-2 affibody)*	- Higher specificity for HER-2^+^ BC cells - Higher r2 relaxivity, good dispersion and biocompatibility	in vitro—in vivo	[137]
**In Vivo Fluorescence Imaging**	MnCuInS/ZnS@BSA-Anti-HER-2 bioconjugates *(Anti-HER-2 antibody, Sino Biological Inc., Beijing, China)*	- Good biocompatibility, low cytotoxicity, high colloidal stability - Higher selectivity of HER-2^+^ cancer cells	in vitro	[140]
	Anti-HER-2-QD-antiboy conjugate *(Anti-HER-2 antibody, Invitrogen, Carlsbad, CA, USA)*	- Localization of HER-2 receptors in both fixed and live cancer cells - Good biocompatibility	in vitro	[141]
	Peptide Nanoprobes	- Two novel peptides YLFFVFER (H6) and KLRLEWNR (H10) show good specificity toward HER-2 - Lower toxicity and good biocompatibility	in vivo—ex vivo	[142]
	*sdAb*-HER-2-QD	- Higher specificity and sensitivity - Detection of micrometastases and disseminated tumor cells	ex vivo	[143]
**Nuclear Medicine**	[^18^F]FB-anti-HER-2 nanobody *(In-house recombinant anti-HER-2 nb 2Rs15d)*	- Excellent targeting properties and specificity for HER-2 Higher tumor-to-blood ratio - Non-competitive nature with trastuzumab for binding to the HER-2 receptor	in vivo	[144]
	^99^mTc-Z*_HER2:2395_*-Cysc (In-house recombinant production)	- High and specific uptake in HER-2^+^ cells - Reduced off-target liver uptake	in vitro—in vivo	[145]
	^99^mTc-radiolabeled nanosilica system, functionalized with a *TZ* half-chain (Genentech, South San Francisco, CA, USA)	- Increased selective accumulation within the HER-2^+^ cells - Enhanced (but not significantly) tumor targeting for functionalized NPs 4h post injection - Good safety	in vitro—ex vivo	[148]
**Multimodal Hybrid Approaches**	*TZ*-conjugated Lipo[MNP@m-SiO2]-HER-2_Ab_ (Genentech, Inc., South San Francisco, CA, USA)	- Biological stability - Higher specificity to HER-2/neu-overexpressing BC cells	In vitro	[151]
	*DARPin G3* coated fluorecently labelled SPIONs	- Higher binding and improved cytotoxicity in SBKR-3 cells - Higher MRI contrast in vivo after systemic administration of SPIONs	In vitro—in vivo	[104]
	HS-Fe-PEG-HER-2 *(Anti-HER-2 antibody, Abcam, Cambridge, UK)*	- Good physical properties and biosafety, low-cytotoxicity - Dual-mode US–MR-specific imaging agent - Higher specificity to HER-2^+^ BC cells	in vitro—in vivo	[153]
	G5-AuNP-Gd-*TZ* *(Genetech, San Francisco, CA, USA)*	- Higher specificity to HER-2^+^ cells - Efficient targeting of HER-2^+^ breast tumors - Enhanced MRI signal and CT resolution	in vitro—in vivo	[154]
	SPIONs-Cy-PEG-scFv *(Recombinant scFv 4D5-Cys)*	- Higher affinity and specificity versus HER-2 overexpressing cells/tumors	In vitro—in vivo	[136]
	^89^Zr-DFO-scFv-PEG-Cy5-C’ dots *(In-house recombinant anti-HER-2 scFv fragments-TZ)*	- Specific accumulation into HER-2^+^ tumors	in vitro—in vivo	[155]
**Inorganic NPs**	Pegylated iron oxide NPs conjugated with anti-HER-2 antibodies *(Herceptin, F. Hoffmann-La Roche Ltd., Basel, Switzerland)*	- Simplicity and accuracy of method - The method has a low detection limit with excellent sensitivity	in vitro	[157]
	Dx-M and As-M were conjugated with a monoclonal scFv *(In-house recombinant* *scFvs)*	- As-M NPs were more efficient in scFv immobilization than Dx-M NPs - chemical modification with aminosilane improved the HER-2 detection. - As-M were also detectable by Raman-Spectroscopy	in vitro	[158]
	Anti-HER-2 antibody-conjugated silver nanoparticles *(Anti-HER-2 antibody, Fuzhou Maxim Biotech, Inc., Fuzhou, China)*	- High sensitivity for targeting HER-2 - Easy fabrication, high SERS sensitivity and biocompatibility	in vitro	[159]
	BSA-AuNCs-LPs-anti-HER-2 *(Anti-HER-2 antibody, R&D Systems, Minneapolis, MN, USA)*	- Higher sensitivity and selectivity of the HER-2^+^ BC cell lines/tissue - Simple and economic approach: colorimetric “readout”	in vitro	[160]
	anti HER-2 antibody-biotin conjugate labelled with commercially available QDs (QD525)	- A Sensitive reporter of HER-2 expression in BC cells and tissues - Superiority over conventional fluorophores in terms of resistance to photobleaching - Higher fluorescent intensity, higher staining index and lower minimum detection limit	in vitro	[161]
**Aptamers**	HeA2_1 and HeA2_3	- Higher specificity to HER-2-overexpressing cells and HER-2^+^ tumor tissue samples - Inhibitory effect on cancer cell growth and viability related to the aptamer’s specificity for HER-2	in vitro—ex vivo	[162]
	HB5	- Specific binding to HER-2 protein and HER-2^+^ BC cells	in vitro	[163]
	APlaS to detect ECD-HER-2 protein	- Higher sensitivity and selectivity	in vitro	[164]
**Antibodies**	HER2-DOX-SPIOs@PLGA@A *(Herceptin)*	- High targeting of HER-2^+^ cells - Targeted drug delivery combined with photothermal-responsive drug release - Good biosafety in vivo and good antitumor effect	in vitro—in vivo	[165]
	^99^mTc-SiNPs-*TZ*/DOX-SiNPs-*TZ*	- Good specificity to HER-2^+^ BC lesions - Higher uptake in HER-2 overexpressing cells - DOX-SiNPs-TZ NPs are able to deliver DOX at tumor site: tumor growth inhibition	in vitro—ex vivo—in vivo	[150]
	IONP/DOX-MFNC *(Herceptin, Chonnam National University* *Hwasun Hospital)*	- Higher cellular uptake and stronger cytotoxicity - Higher tumor uptake - Enhanced therapeutic effects via HER-2-mediated selectivity: tumor regression	in vitro—in vivo	[166]

## 8. Conclusions

HER-2^+^ BC is a pathology much studied by nanotechnologists who have developed many nanosystems aimed at improving the treatment and diagnosis of cancer. Starting from therapeutic antibodies currently used in BC therapy, many targeted NPs have been developed. Therapeutic antibodies and their derivatives have been used to functionalize different kinds of NPs in an attempt to drive tumor recognition, NP accumulation, and internalization, resulting in increased performance as diagnostic devices, and with therapeutic properties. 

NPs also allow the delivery of hydrophobic molecules, due to their encapsulation in appropriate nanosystems, improving their bioavailability and hence favoring their use as new therapeutic agents with improved therapeutic and imaging properties. Indeed, by modifying NP characteristics, the pharmacokinetics of injected molecules can be enhanced and their blood half-life extended. The toxicity of parenterally administered drugs can be reduced by enhancing drug accumulation i the target tissue and reducing off-target effects. Moreover, a single NP can be loaded with several drug molecules, allowing testing of different therapeutic combinations to improve BC management and reduce the onset of chemoresistance. In parallel, NPs can be loaded with several contrast agents and used as highly sensitive tools for early cancer diagnosis, as active moieties for radiation therapy and as theranostic tools if co-loaded with active drugs. Moreover, as the cellular uptake mechanisms of encapsulated drugs are completely different from those of free drugs, this has been shown to significantly reduce the evolution of resistance mechanisms. 

Among all the nanotechnological solutions proposed so far, a preferred candidate NP, able to be used as a safe and active targeted nanodrug for HER-2^+^ BC, has not emerged yet. This is due to several factors that need further investigation, including difficulties in the translation of the results from in vitro to in vivo experiments in murine models of cancer, rapid clearance from the bloodstream and liver sequestration, intrinsic toxicity of the material used to prepare the NPs, and low reproducibility and high production costs.

We believe that in the effort of developing NPs for the management of HER-2^+^ BC, the focus on the translational potential of the agents should always be central at the very beginning of nanodrug development. 

## Abbreviations

BC	Breast cancer
HER-2^+^ BC	Human epidermal growth factor receptor-2 overexpressing breast cancer
NPs	Nanoparticles
EGFR	Epidermal growth factor receptor
AKT	Protein kinase B, PKB
PI3K	Phosphoinositide 3-kinase
MAPK	Mitogen-activated protein kinase
IHC	Immunohistochemistry
FISH	Fluorescence in situ hybridization
mAb	Monoclonal antibodies
TZ	Trastuzumab
DFS	Disease-free survival
OS	Overall survival
ADCC	Antibody-Dependent Cellular Cytotoxicity
T-DM1, Kadcyla^®^	Trastuzumab emtansine
ADCs	Antibody-drug conjugates, anticancer drugs
PFS	Progression-free survival
PTEN	Phosphatase and tensin homolog
TKIs	Tyrosine kinase inhibitors
pCR	Pathological complete response
TILs	Tumor-infiltrating lymphocytes
PEI	Polyethylenimine
PLGA	Poly (D,L-lactide-co-glycolide)
sdAb	Single-domain antibodies
VH	Human immunoglobulin heavy chain
Fab	Antibody fragments
TmAb	Fab obtained by papain cleavage of TZ
PmAb	Fab obtained by papain cleavage of panitumumab
scFv	Single chain variable fragments
DOX	Doxorubicin
PTX	Paclitaxel
PCI	Photochemical Internalization
siRNA	Short interfering RNA
PTT	Photothermal therapy
GNRs	Gold nanorods
AuNPs	Gold nanoparticles
PK	Pharmacokinetic
XBP1	X-box binding protein 1
PDT	Photodynamic therapy
Hapt	Aptamer
IHC	Immunohistochemistry
CISH	Chromogenic in-situ hybridization
CT	Computerized tomography
PET	Positron emission tomography
SPECT	Single photon emission computed tomography
US	Ultrasounds
MRI	Magnetic resonance imaging
SPIONs	Super paramagnetic iron oxide nanoparticles
FGS	Fluorescence guided surgery
QDs	Quantum dots
Cy	Cyanine
BSA	Bovine serum albumin
EPR	Enhanced Permeation and Retention
PAMAM	Polyamidoamine
SERS	Surface-enhanced Raman scattering
SELEX	Systematic evolution of ligands by exponential enrichment

## References

[1] Siegel R.L., Miller K.D., Jemal A. (2020). Cancer Statistics, 2020. CA Cancer J. Clin..

[2] Sung H., Ferlay J., Siegel R.L., Laversanne M., Soerjomataram I., Jemal A., Bray F. (2021). Global Cancer Statistics 2020: GLOBOCAN Estimates of Incidence and Mortality Worldwide for 36 Cancers in 185 Countries. CA Cancer J. Clin..

[3] Howlader N., Cronin K.A., Kurian A.W., Andridge R. (2018). Differences in Breast Cancer Survival by Molecular Subtypes in the United States. Cancer Epidemiol. Biomark. Prev..

[4] Mittendorf E.A., Wu Y., Scaltriti M., Meric-Bernstam F., Hunt K.K., Dawood S., Esteva F.J., Buzdar A.U., Chen H., Eksambi S. (2009). Loss of HER2 Amplification Following Trastuzumab-Based Neoadjuvant Systemic Therapy and Survival Outcomes. Clin. Cancer Res..

[5] Niikura N., Liu J., Hayashi N., Mittendorf E.A., Gong Y., Palla S.L., Tokuda Y., Gonzalez-Angulo A.M., Hortobagyi G.N., Ueno N.T. (2012). Loss of Human Epidermal Growth Factor Receptor 2 (HER2) Expression in Metastatic Sites of HER2-Overexpressing Primary Breast Tumors. J. Clin. Oncol..

[6] Mohsin S.K., Weiss H.L., Gutierrez M.C., Chamness G.C., Schiff R., DiGiovanna M.P., Wang C.-X., Hilsenbeck S.G., Osborne C.K., Allred D.C. (2005). Neoadjuvant Trastuzumab Induces Apoptosis in Primary Breast Cancers. J. Clin. Oncol..

[7] Gallardo A., Lerma E., Escuin D., Tibau A., Muñoz J., Ojeda B., Barnadas A., Adrover E., Sánchez-Tejada L., Giner D. (2012). Increased Signalling of EGFR and IGF1R, and Deregulation of PTEN/PI3K/Akt Pathway Are Related with Trastuzumab Resistance in HER2 Breast Carcinomas. Br. J. Cancer.

[8] Arvold N.D., Oh K.S., Niemierko A., Taghian A.G., Lin N.U., Abi-Raad R.F., Sreedhara M., Harris J.R., Alexander B.M. (2012). Brain Metastases after Breast-Conserving Therapy and Systemic Therapy: Incidence and Characteristics by Biologic Subtype. Breast Cancer Res Treat..

[9] Kennecke H., Yerushalmi R., Woods R., Cheang M.C.U., Voduc D., Speers C.H., Nielsen T.O., Gelmon K. (2010). Metastatic Behavior of Breast Cancer Subtypes. J. Clin. Oncol..

[10] Schlessinger J. (2004). Common and Distinct Elements in Cellular Signaling via EGF and FGF Receptors. Science.

[11] Jiang N., Dai Q., Su X., Fu J., Feng X., Peng J. (2020). Role of PI3K/AKT Pathway in Cancer: The Framework of Malignant Behavior. Mol. Biol. Rep..

[12] Ruiz-Saenz A., Dreyer C., Campbell M.R., Steri V., Gulizia N.P., Moasser M.M. (2018). HER2 Amplification in Tumors Activates PI3K/Akt Signaling Independent of HER3. Cancer Res..

[13] Sheen M.R., Marotti J.D., Allegrezza M.J., Rutkowski M., Conejo-Garcia J.R., Fiering S. (2016). Constitutively Activated PI3K Accelerates Tumor Initiation and Modifies Histopathology of Breast Cancer. Oncogenesis.

[14] Dhillon A.S., Hagan S., Rath O., Kolch W. (2007). MAP Kinase Signalling Pathways in Cancer. Oncogene.

[15] Wolff A.C., Hammond M.E.H., Allison K.H., Harvey B.E., Mangu P.B., Bartlett J.M.S., Bilous M., Ellis I.O., Fitzgibbons P., Hanna W. (2018). Human Epidermal Growth Factor Receptor 2 Testing in Breast Cancer: American Society of Clinical Oncology/College of American Pathologists Clinical Practice Guideline Focused Update. Arch. Pathol. Lab. Med..

[16] Cardoso F., Senkus E., Costa A., Papadopoulos E., Aapro M., André F., Harbeck N., Aguilar Lopez B., Barrios C.H., Bergh J. (2018). 4th ESO–ESMO International Consensus Guidelines for Advanced Breast Cancer (ABC 4). Ann. Oncol..

[17] Swain S.M., Miles D., Kim S.-B., Im Y.-H., Im S.-A., Semiglazov V., Ciruelos E., Schneeweiss A., Loi S., Monturus E. (2020). Pertuzumab, Trastuzumab, and Docetaxel for HER2-Positive Metastatic Breast Cancer (CLEOPATRA): End-of-Study Results from a Double-Blind, Randomised, Placebo-Controlled, Phase 3 Study. Lancet Oncol..

[18] Cardoso F., Kyriakides S., Ohno S., Penault-Llorca F., Poortmans P., Rubio I.T., Zackrisson S., Senkus E. (2019). Early Breast Cancer: ESMO Clinical Practice Guidelines for Diagnosis, Treatment and Follow-Up. Ann. Oncol..

[19] Slamon D.J., Leyland-Jones B., Shak S., Fuchs H., Paton V., Bajamonde A., Fleming T., Eiermann W., Wolter J., Pegram M. (2001). Use of Chemotherapy plus a Monoclonal Antibody against HER2 for Metastatic Breast Cancer That Overexpresses HER2. N. Engl. J. Med.

[20] Piccart-Gebhart M.J., Procter M., Leyland-Jones B., Goldhirsch A., Untch M., Smith I., Gianni L., Baselga J., Bell R., Jackisch C. (2005). Trastuzumab after Adjuvant Chemotherapy in HER2-Positive Breast Cancer. N. Engl. J. Med.

[21] Smith I., Procter M., Gelber R.D., Guillaume S., Feyereislova A., Dowsett M., Goldhirsch A., Untch M., Mariani G., Baselga J. (2007). 2-Year Follow-up of Trastuzumab after Adjuvant Chemotherapy in HER2-Positive Breast Cancer: A Randomised Controlled Trial. Lancet.

[22] Vogel C.L., Cobleigh M.A., Tripathy D., Gutheil J.C., Harris L.N., Fehrenbacher L., Slamon D.J., Murphy M., Novotny W.F., Burchmore M. (2002). Efficacy and Safety of Trastuzumab as a Single Agent in First-Line Treatment of *HER2*-Overexpressing Metastatic Breast Cancer. J. Clin. Oncol..

[23] Meng Y., Zheng L., Yang Y., Wang H., Dong J., Wang C., Zhang Y., Yu X., Wang L., Xia T. (2016). A Monoclonal Antibody Targeting ErbB2 Domain III Inhibits ErbB2 Signaling and Suppresses the Growth of ErbB2-Overexpressing Breast Tumors. Oncogenesis.

[24] Zhu H., Zhang G., Wang Y., Xu N., He S., Zhang W., Chen M., Liu M., Quan L., Bai J. (2010). Inhibition of ErbB2 by Herceptin Reduces Survivin Expression via the ErbB2-β-Catenin/TCF4-Survivin Pathway in ErbB2-Overexpressed Breast Cancer Cells. Cancer Sci..

[25] Davis N.M., Sokolosky M., Stadelman K., Abrams S.L., Libra M., Candido S., Nicoletti F., Polesel J., Maestro R., D’Assoro A. (2014). Deregulation of the EGFR/PI3K/PTEN/Akt/MTORC1 Pathway in Breast Cancer: Possibilities for Therapeutic Intervention. Oncotarget.

[26] Fiszman G.L., Jasnis M.A. (2011). Molecular Mechanisms of Trastuzumab Resistance in HER2 Overexpressing Breast Cancer. Int. J. Breast Cancer.

[27] Capietto A.-H., Martinet L., Fournié J.-J. (2011). Stimulated Γδ T Cells Increase the In Vivo Efficacy of Trastuzumab in HER-2^+^ Breast Cancer. J. Immunol..

[28] Nagata Y., Lan K.-H., Zhou X., Tan M., Esteva F.J., Sahin A.A., Klos K.S., Li P., Monia B.P., Nguyen N.T. (2004). PTEN Activation Contributes to Tumor Inhibition by Trastuzumab, and Loss of PTEN Predicts Trastuzumab Resistance in Patients. Cancer Cell.

[29] Nahta R., Esteva F.J. (2006). HER2 Therapy: Molecular Mechanisms of Trastuzumab Resistance. Breast Cancer Res..

[30] Scaltriti M., Rojo F., Ocana A., Anido J., Guzman M., Cortes J., Di Cosimo S., Matias-Guiu X., Ramon y Cajal S., Arribas J. (2007). Expression of P95HER2, a Truncated Form of the HER2 Receptor, and Response to Anti-HER2 Therapies in Breast Cancer. JNCI J. Natl. Cancer Inst..

[31] Hurvitz S.A., Martin M., Jung K.H., Huang C.-S., Harbeck N., Valero V., Stroyakovskiy D., Wildiers H., Campone M., Boileau J.-F. (2019). Neoadjuvant Trastuzumab Emtansine and Pertuzumab in Human Epidermal Growth Factor Receptor 2–Positive Breast Cancer: Three-Year Outcomes from the Phase III KRISTINE Study. J. Clin. Oncol..

[32] Slamon D., Eiermann W., Robert N., Pienkowski T., Martin M., Press M., Mackey J., Glaspy J., Chan A., Pawlicki M. (2011). Adjuvant Trastuzumab in HER2-Positive Breast Cancer. N. Engl. J. Med..

[33] Rugo H.S., Im S.-A., Cardoso F., Cortés J., Curigliano G., Musolino A., Pegram M.D., Wright G.S., Saura C., Escrivá-de-Romaní S. (2021). Efficacy of Margetuximab vs Trastuzumab in Patients with Pretreated ERBB2-Positive Advanced Breast Cancer: A Phase 3 Randomized Clinical Trial. JAMA Oncol..

[34] Lewis Phillips G.D., Li G., Dugger D.L., Crocker L.M., Parsons K.L., Mai E., Blättler W.A., Lambert J.M., Chari R.V.J., Lutz R.J. (2008). Targeting HER2-Positive Breast Cancer with Trastuzumab-DM1, an Antibody–Cytotoxic Drug Conjugate. Cancer Res..

[35] Diéras V., Miles D., Verma S., Pegram M., Welslau M., Baselga J., Krop I.E., Blackwell K., Hoersch S., Xu J. (2017). Trastuzumab Emtansine versus Capecitabine plus Lapatinib in Patients with Previously Treated HER2-Positive Advanced Breast Cancer (EMILIA): A Descriptive Analysis of Final Overall Survival Results from a Randomised, Open-Label, Phase 3 Trial. Lancet Oncol..

[36] Loibl S., Majewski I., Guarneri V., Nekljudova V., Holmes E., Bria E., Denkert C., Schem C., Sotiriou C., Loi S. (2016). PIK3CA Mutations Are Associated with Reduced Pathological Complete Response Rates in Primary HER2-Positive Breast Cancer: Pooled Analysis of 967 Patients from Five Prospective Trials Investigating Lapatinib and Trastuzumab. Ann. Oncol..

[37] Molina M.A., Codony-Servat J., Albanell J., Rojo F., Arribas J., Baselga J. (2001). Trastuzumab (Herceptin), a Humanized Anti-Her2 Receptor Monoclonal Antibody, Inhibits Basal and Activated Her2 Ectodomain Cleavage in Breast Cancer Cells. Cancer Res..

[38] Ghosh R., Narasanna A., Wang S.E., Liu S., Chakrabarty A., Balko J.M., González-Angulo A.M., Mills G.B., Penuel E., Winslow J. (2011). Trastuzumab Has Preferential Activity against Breast Cancers Driven by HER2 Homodimers. Cancer Res..

[39] Ríos-Luci C., García-Alonso S., Díaz-Rodríguez E., Nadal-Serrano M., Arribas J., Ocaña A., Pandiella A. (2017). Resistance to the Antibody–Drug Conjugate T-DM1 Is Based in a Reduction in Lysosomal Proteolytic Activity. Cancer Res..

[40] Sauveur J., Matera E.-L., Chettab K., Valet P., Guitton J., Savina A., Dumontet C. (2018). Esophageal Cancer Cells Resistant to T-DM1 Display Alterations in Cell Adhesion and the Prostaglandin Pathway. Oncotarget.

[41] Columbus G. (2019). Trastuzumab Deruxtecan Receives Accelerated Approval by FDA for HER2+ Breast Cancer. https://www.targetedonc.com/view/trastuzumab-deruxtecan-receives-accelerated-approval-by-fda-for-her2-breast-cancer.

[42] Rinnerthaler G., Gampenrieder S., Greil R. (2019). HER2 Directed Antibody-Drug-Conjugates beyond T-DM1 in Breast Cancer. Int. J. Mol. Sci..

[43] Martínez-Sáez O., Chic N., Pascual T., Adamo B., Vidal M., González-Farré B., Sanfeliu E., Schettini F., Conte B., Brasó-Maristany F. (2020). Frequency and Spectrum of PIK3CA Somatic Mutations in Breast Cancer. Breast Cancer Res..

[44] Verret B., Cortes J., Bachelot T., Andre F., Arnedos M. (2019). Efficacy of PI3K Inhibitors in Advanced Breast Cancer. Ann. Oncol..

[45] André F., Hurvitz S., Fasolo A., Tseng L.-M., Jerusalem G., Wilks S., O’Regan R., Isaacs C., Toi M., Burris H.A. (2016). Molecular Alterations and Everolimus Efficacy in Human Epidermal Growth Factor Receptor 2–Overexpressing Metastatic Breast Cancers: Combined Exploratory Biomarker Analysis From BOLERO-1 and BOLERO-3. J. Clin. Oncol..

[46] Segovia-Mendoza M., González-González M.E., Barrera D., Díaz L., García-Becerra R. (2015). Efficacy and Mechanism of Action of the Tyrosine Kinase Inhibitors Gefitinib, Lapatinib and Neratinib in the Treatment of HER2-Positive Breast Cancer: Preclinical and Clinical Evidence. Am. J. Cancer Res..

[47] Esparís-Ogando A., Montero J., Arribas J., Ocaña A., Pandiella A. (2016). Targeting the EGF/HER Ligand-Receptor System in Cancer. Curr. Pharm. Des..

[48] Chan A., Delaloge S., Holmes F.A., Moy B., Iwata H., Harvey V.J., Robert N.J., Silovski T., Gokmen E., Von Minckwitz G. (2016). Neratinib after Trastuzumab-Based Adjuvant Therapy in Patients with HER2-Positive Breast Cancer (ExteNET): A Multicentre, Randomised, Double-Blind, Placebo-Controlled, Phase 3 Trial. Lancet Oncol..

[49] Singh H., Walker A.J., Amiri-Kordestani L., Cheng J., Tang S., Balcazar P., Barnett-Ringgold K., Palmby T.R., Cao X., Zheng N. (2018). U.S. Food and Drug Administration Approval: Neratinib for the Extended Adjuvant Treatment of Early-Stage HER2-Positive Breast Cancer. Clin. Cancer Res..

[50] Leo C.P., Hentschel B., Szucs T.D., Leo C. (2020). FDA and EMA Approvals of New Breast Cancer Drugs—A Comparative Regulatory Analysis. Cancers.

[51] De Azambuja E., Holmes A.P., Piccart-Gebhart M., Holmes E., Di Cosimo S., Swaby R.F., Untch M., Jackisch C., Lang I., Smith I. (2014). Lapatinib with Trastuzumab for HER2-Positive Early Breast Cancer (NeoALTTO): Survival Outcomes of a Randomised, Open-Label, Multicentre, Phase 3 Trial and Their Association with Pathological Complete Response. Lancet Oncol..

[52] Savas P., Salgado R., Denkert C., Sotiriou C., Darcy P.K., Smyth M.J., Loi S. (2016). Clinical Relevance of Host Immunity in Breast Cancer: From TILs to the Clinic. Nat. Rev. Clin. Oncol..

[53] Hou Y., Nitta H., Wei L., Banks P.M., Lustberg M., Wesolowski R., Ramaswamy B., Parwani A.V., Li Z. (2018). PD-L1 Expression and CD8-Positive T Cells Are Associated with Favorable Survival in HER2-Positive Invasive Breast Cancer. Breast J..

[54] Poole R.M. (2014). Pembrolizumab: First Global Approval. Drugs.

[55] Markham A. (2016). Atezolizumab: First Global Approval. Drugs.

[56] Guo L., Zhang H., Chen B. (2017). Nivolumab as Programmed Death-1 (PD-1) Inhibitor for Targeted Immunotherapy in Tumor. J. Cancer.

[57] Nieto C., Vega M.A., Martín del Valle E.M. (2020). Trastuzumab: More than a Guide in HER2-Positive Cancer Nanomedicine. Nanomaterials.

[58] White B.E., White M.K., Adhvaryu H., Makhoul I., Nima Z.A., Biris A.S., Ali N. (2020). Nanotechnology Approaches to Addressing HER2-Positive Breast Cancer. Cancer Nano.

[59] Kumar M., Rajnikanth P.S. (2020). A Mini-Review on HER2 Positive Breast Cancer and Its Metastasis: Resistance and Treatment Strategies. Curr. Nanomed. (Former. Recent Pat. Nanomed.).

[60] Marques A.C., Costa P.J., Velho S., Amaral M.H. (2020). Functionalizing Nanoparticles with Cancer-Targeting Antibodies: A Comparison of Strategies. J. Control. Release.

[61] Juan A., Cimas F.J., Bravo I., Pandiella A., Ocaña A., Alonso-Moreno C. (2020). An Overview of Antibody Conjugated Polymeric Nanoparticles for Breast Cancer Therapy. Pharmaceutics.

[62] Lin X., O’Reilly Beringhs A., Lu X. (2021). Applications of Nanoparticle-Antibody Conjugates in Immunoassays and Tumor Imaging. AAPS J..

[63] Bloise N., Massironi A., Della Pina C., Alongi J., Siciliani S., Manfredi A., Biggiogera M., Rossi M., Ferruti P., Ranucci E. (2020). Extra-Small Gold Nanospheres Decorated with a Thiol Functionalized Biodegradable and Biocompatible Linear Polyamidoamine as Nanovectors of Anticancer Molecules. Front. Bioeng. Biotechnol..

[64] Cai Z., Chattopadhyay N., Yang K., Kwon Y.L., Yook S., Pignol J.-P., Reilly R.M. (2016). 111In-Labeled Trastuzumab-Modified Gold Nanoparticles Are Cytotoxic in Vitro to HER2-Positive Breast Cancer Cells and Arrest Tumor Growth in Vivo in Athymic Mice after Intratumoral Injection. Nucl. Med. Biol..

[65] Dziawer Ł., Majkowska-Pilip A., Gaweł D., Godlewska M., Pruszyński M., Jastrzębski J., Wąs B., Bilewicz A. (2019). Trastuzumab-Modified Gold Nanoparticles Labeled with 211At as a Prospective Tool for Local Treatment of HER2-Positive Breast Cancer. Nanomaterials.

[66] Kang X., Guo X., Niu X., An W., Li S., Liu Z., Yang Y., Wang N., Jiang Q., Yan C. (2017). Photothermal Therapeutic Application of Gold Nanorods-Porphyrin-Trastuzumab Complexes in HER2-Positive Breast Cancer. Sci Rep..

[67] Zhang X., Liu J., Li X., Li F., Lee R.J., Sun F., Li Y., Liu Z., Teng L. (2019). Trastuzumab-Coated Nanoparticles Loaded with Docetaxel for Breast Cancer Therapy. Dose-Response.

[68] Yu K., Zhao J., Zhang Z., Gao Y., Zhou Y., Teng L., Li Y. (2016). Enhanced Delivery of Paclitaxel Using Electrostatically-Conjugated Herceptin-Bearing PEI/PLGA Nanoparticles against HER-Positive Breast Cancer Cells. Int. J. Pharm..

[69] Naruphontjirakul P., Viravaidya-Pasuwat K. (2019). Development of Anti-HER2-Targeted Doxorubicin–Core-Shell Chitosan Nanoparticles for the Treatment of Human Breast Cancer. Int. J. Nanomed..

[70] Peng J., Chen J., Xie F., Bao W., Xu H., Wang H., Xu Y., Du Z. (2019). Herceptin-Conjugated Paclitaxel Loaded PCL-PEG Worm-like Nanocrystal Micelles for the Combinatorial Treatment of HER2-Positive Breast Cancer. Biomaterials.

[71] Varshosaz J., Ghassami E., Noorbakhsh A., Minaiyan M., Jahanian-Najafabadi A. (2019). Trastuzumab-conjugated Nanoparticles Composed of Poly(Butylene Adipate-*Co*-butylene Terephthalate) Prepared by Electrospraying Technique for Targeted Delivery of Docetaxel. IET Nanobiotechnol..

[72] Domínguez-Ríos R., Sánchez-Ramírez D.R., Ruiz-Saray K., Oceguera-Basurto P.E., Almada M., Juárez J., Zepeda-Moreno A., del Toro-Arreola A., Topete A., Daneri-Navarro A. (2019). Cisplatin-Loaded PLGA Nanoparticles for HER2 Targeted Ovarian Cancer Therapy. Colloids Surf. B Biointerfaces.

[73] Cędrowska E., Pruszyński M., Gawęda W., Żuk M., Krysiński P., Bruchertseifer F., Morgenstern A., Karageorgou M.-A., Bouziotis P., Bilewicz A. (2020). Trastuzumab Conjugated Superparamagnetic Iron Oxide Nanoparticles Labeled with 225Ac as a Perspective Tool for Combined α-Radioimmunotherapy and Magnetic Hyperthermia of HER2-Positive Breast Cancer. Molecules.

[74] Korangath P., Barnett J.D., Sharma A., Henderson E.T., Stewart J., Yu S.-H., Kandala S.K., Yang C.-T., Caserto J.S., Hedayati M. (2020). Nanoparticle Interactions with Immune Cells Dominate Tumor Retention and Induce T Cell–Mediated Tumor Suppression in Models of Breast Cancer. Sci. Adv..

[75] Ko N.R., Van S.Y., Hong S.H., Kim S.-Y., Kim M., Lee J.S., Lee S.J., Lee Y., Kwon I.K., Oh S.J. (2020). Dual PH- and GSH-Responsive Degradable PEGylated Graphene Quantum Dot-Based Nanoparticles for Enhanced HER2-Positive Breast Cancer Therapy. Nanomaterials.

[76] Shu M., Gao F., Yu C., Zeng M., He G., Wu Y., Su Y., Hu N., Zhou Z., Yang Z. (2020). Dual-Targeted Therapy in HER2-Positive Breast Cancer Cells with the Combination of Carbon Dots/HER3 SiRNA and Trastuzumab. Nanotechnology.

[77] Tanaka S., Matsunami N., Morishima H., Oda N., Takashima T., Noda S., Kashiwagi S., Tauchi Y., Asano Y., Kimura K. (2019). De-Escalated Neoadjuvant Therapy with Nanoparticle Albumin-Bound Paclitaxel and Trastuzumab for Low-Risk Pure HER2 Breast Cancer. Cancer Chemother Pharm..

[78] Duan D., Wang A., Ni L., Zhang L., Yan X., Jiang Y., Mu H., Wu Z., Sun K., Li Y. (2018). Nanoparticle Interactions with Immune Cells Dominate Tumor Retention and Induce T Cell–Mediated Tumor Suppression in Models of Breast Cancer. Int. J. Nanomed..

[79] Kim B., Shin J., Wu J., Omstead D.T., Kiziltepe T., Littlepage L.E., Bilgicer B. (2020). Engineering Peptide-Targeted Liposomal Nanoparticles Optimized for Improved Selectivity for HER2-Positive Breast Cancer Cells to Achieve Enhanced in Vivo Efficacy. J. Control. Release.

[80] Houdaihed L., Evans J.C., Allen C. (2020). Dual-Targeted Delivery of Nanoparticles Encapsulating Paclitaxel and Everolimus: A Novel Strategy to Overcome Breast Cancer Receptor Heterogeneity. Pharm. Res..

[81] Vorotnikov Y.A., Novikova E.D., Solovieva A.O., Shanshin D.V., Tsygankova A.R., Shcherbakov D.N., Efremova O.A., Shestopalov M.A. (2020). Single-Domain Antibody C7b for Address Delivery of Nanoparticles to HER2-Positive Cancers. Nanoscale.

[82] Holliger P., Hudson P.J. (2005). Engineered Antibody Fragments and the Rise of Single Domains. Nat. Biotechnol..

[83] Van Audenhove I., Gettemans J. (2016). Nanobodies as Versatile Tools to Understand, Diagnose, Visualize and Treat Cancer. EBioMedicine.

[84] Martínez-Jothar L., Beztsinna N., van Nostrum C.F., Hennink W.E., Oliveira S. (2019). Selective Cytotoxicity to HER2 Positive Breast Cancer Cells by Saporin-Loaded Nanobody-Targeted Polymeric Nanoparticles in Combination with Photochemical Internalization. Mol. Pharm..

[85] Li M., Dong J., Cheng F., Li C., Wang H., Sun T., He W., Wang Q. (2021). Controlling Conjugated Antibodies at the Molecular Level for Active Targeting Nanoparticles toward HER2-Positive Cancer Cells. Mol. Pharm..

[86] Shi X., Cheng Q., Hou T., Han M., Smbatyan G., Lang J.E., Epstein A.L., Lenz H.-J., Zhang Y. (2020). Genetically Engineered Cell-Derived Nanoparticles for Targeted Breast Cancer Immunotherapy. Mol. Ther..

[87] Okarvi S.M., AlJammaz I. (2019). Development of the Tumor-Specific Antigen-Derived Synthetic Peptides as Potential Candidates for Targeting Breast and Other Possible Human Carcinomas. Molecules.

[88] Ding H., Gangalum P.R., Galstyan A., Fox I., Patil R., Hubbard P., Murali R., Ljubimova J.Y., Holler E. (2017). HER2-Positive Breast Cancer Targeting and Treatment by a Peptide-Conjugated Mini Nanodrug. Nanomed. Nanotechnol. Biol. Med..

[89] Mu Q., Kievit F.M., Kant R.J., Lin G., Jeon M., Zhang M. (2015). Anti-HER2/Neu Peptide-Conjugated Iron Oxide Nanoparticles for Targeted Delivery of Paclitaxel to Breast Cancer Cells. Nanoscale.

[90] Zhang L., Jing D., Jiang N., Rojalin T., Baehr C.M., Zhang D., Xiao W., Wu Y., Cong Z., Li J.J. (2020). Transformable Peptide Nanoparticles Arrest HER2 Signalling and Cause Cancer Cell Death in Vivo. Nat. Nanotechnol..

[91] Zhu G., Chen X. (2018). Aptamer-Based Targeted Therapy. Adv. Drug Deliv. Rev..

[92] Saleh T., Soudi T., Shojaosadati S.A. (2019). Aptamer Functionalized Curcumin-Loaded Human Serum Albumin (HSA) Nanoparticles for Targeted Delivery to HER-2 Positive Breast Cancer Cells. Int. J. Biol. Macromol..

[93] Lee H., Dam D.H.M., Ha J.W., Yue J., Odom T.W. (2015). Enhanced Human Epidermal Growth Factor Receptor 2 Degradation in Breast Cancer Cells by Lysosome-Targeting Gold Nanoconstructs. ACS Nano.

[94] Shen Y., Li M., Liu T., Liu J., Xie Y., Zhang J., Xu S., Liu H. (2019). A Dual-Functional HER2 Aptamer-Conjugated, PH-Activated Mesoporous Silica Nanocarrier-Based Drug Delivery System Provides In Vitro Synergistic Cytotoxicity in HER2-Positive Breast Cancer Cells. Int. J. Nanomed..

[95] Ma W., Zhan Y., Zhang Y., Shao X., Xie X., Mao C., Cui W., Li Q., Shi J., Li J. (2019). An Intelligent DNA Nanorobot with in Vitro Enhanced Protein Lysosomal Degradation of HER2. Nano Lett..

[96] Bennett V., Baines A.J. (2001). Spectrin and Ankyrin-Based Pathways: Metazoan Inventions for Integrating Cells into Tissues. Physiol. Rev..

[97] Binz H.K., Amstutz P., Plückthun A. (2005). Engineering Novel Binding Proteins from Nonimmunoglobulin Domains. Nat. Biotechnol..

[98] Stumpp M.T., Dawson K.M., Binz H.K. (2020). Beyond Antibodies: The DARPin^®^ Drug Platform. BioDrugs.

[99] Moisseiev E., Loewenstein A. (2020). Abicipar Pegol—A Novel Anti-VEGF Therapy with a Long Duration of Action. Eye.

[100] Zahnd C., Pecorari F., Straumann N., Wyler E., Plückthun A. (2006). Selection and Characterization of Her2 Binding-Designed Ankyrin Repeat Proteins. J. Biol. Chem..

[101] Theurillat J.-P., Dreier B., Nagy-Davidescu G., Seifert B., Behnke S., Zürrer-Härdi U., Ingold F., Plückthun A., Moch H. (2010). Designed Ankyrin Repeat Proteins: A Novel Tool for Testing Epidermal Growth Factor Receptor 2 Expression in Breast Cancer. Mod. Pathol..

[102] Zahnd C., Kawe M., Stumpp M.T., de Pasquale C., Tamaskovic R., Nagy-Davidescu G., Dreier B., Schibli R., Binz H.K., Waibel R. (2010). Efficient Tumor Targeting with High-Affinity Designed Ankyrin Repeat Proteins: Effects of Affinity and Molecular Size. Cancer Res..

[103] Shipunova V.O., Kotelnikova P.A., Aghayeva U.F., Stremovskiy O.A., Novikov I.A., Schulga A.A., Nikitin M.P., Deyev S.M. (2019). Self-Assembling Nanoparticles Biofunctionalized with Magnetite-Binding Protein for the Targeted Delivery to HER2/Neu Overexpressing Cancer Cells. J. Magn. Magn. Mater..

[104] Li D.-L., Tan J.-E., Tian Y., Huang S., Sun P.-H., Wang M., Han Y.-J., Li H.-S., Wu H.-B., Zhang X.-M. (2017). Multifunctional Superparamagnetic Nanoparticles Conjugated with Fluorescein-Labeled Designed Ankyrin Repeat Protein as an Efficient HER2-Targeted Probe in Breast Cancer. Biomaterials.

[105] Guryev E.L., Shilyagina N.Y., Kostyuk A.B., Sencha L.M., Balalaeva I.V., Vodeneev V.A., Kutova O.M., Lyubeshkin A.V., Yakubovskaya R.I., Pankratov A.A. (2019). Preclinical Study of Biofunctional Polymer-Coated Upconversion Nanoparticles. Toxicol. Sci..

[106] Dong Y., Li W., Gu Z., Xing R., Ma Y., Zhang Q., Liu Z. (2019). Inhibition of HER2-Positive Breast Cancer Growth by Blocking the HER2 Signaling Pathway with HER2-Glycan-Imprinted Nanoparticles. Angew. Chem. Int. Ed..

[107] Yezhelyev M.V., Gao X., Xing Y., Al-Hajj A., Nie S., O’Regan R.M. (2006). Emerging Use of Nanoparticles in Diagnosis and Treatment of Breast Cancer. Lancet Oncol..

[108] Zahmatkeshan M., Gheybi F., Rezayat S.M., Jaafari M.R. (2016). Improved Drug Delivery and Therapeutic Efficacy of PEgylated Liposomal Doxorubicin by Targeting Anti-HER2 Peptide in Murine Breast Tumor Model. Eur. J. Pharm. Sci..

[109] Akhtari J., Rezayat S.M., Teymouri M., Alavizadeh S.H., Gheybi F., Badiee A., Jaafari M.R. (2016). Targeting, Bio Distributive and Tumor Growth Inhibiting Characterization of Anti-HER2 Affibody Coupling to Liposomal Doxorubicin Using BALB/c Mice Bearing TUBO Tumors. Int. J. Pharm..

[110] Niza E., Noblejas-López M.D.M., Bravo I., Nieto-Jiménez C., Castro-Osma J.A., Canales-Vázquez J., Lara-Sanchez A., Galán Moya E.M., Burgos M., Ocaña A. (2019). Trastuzumab-Targeted Biodegradable Nanoparticles for Enhanced Delivery of Dasatinib in HER2+ Metastasic Breast Cancer. Nanomaterials.

[111] Wan X., Zheng X., Pang X., Zhang Z., Zhang Q. (2015). Incorporation of Lapatinib into Human Serum Albumin Nanoparticles with Enhanced Anti-Tumor Effects in HER2-Positive Breast Cancer. Colloids Surf. B Biointerfaces.

[112] Dana H., Chalbatani G.M., Mahmoodzadeh H., Karimloo R., Rezaiean O., Moradzadeh A., Mehmandoost N., Moazzen F., Mazraeh A., Marmari V. (2017). Molecular Mechanisms and Biological Functions of SiRNA. Int. J. Biomed. Sci.

[113] Mainini F., Eccles M.R. (2020). Lipid and Polymer-Based Nanoparticle SiRNA Delivery Systems for Cancer Therapy. Molecules.

[114] Gu S., Ngamcherdtrakul W., Reda M., Hu Z., Gray J.W., Yantasee W. (2018). Lack of Acquired Resistance in HER2-Positive Breast Cancer Cells after Long-Term HER2 SiRNA Nanoparticle Treatment. PLoS ONE.

[115] Gu S., Hu Z., Ngamcherdtrakul W., Castro D.J., Morry J., Reda M.M., Gray J.W., Yantasee W. (2016). Therapeutic SiRNA for Drug-Resistant HER2-Positive Breast Cancer. Oncotarget.

[116] Cristofolini T., Dalmina M., Sierra J.A., Silva A.H., Pasa A.A., Pittella F., Creczynski-Pasa T.B. (2020). Multifunctional Hybrid Nanoparticles as Magnetic Delivery Systems for SiRNA Targeting the HER2 Gene in Breast Cancer Cells. Mater. Sci. Eng. C.

[117] Zhang L., Mu C., Zhang T., Wang Y., Wang Y., Fan L., Liu C., Chen H., Shen J., Wei K. (2020). Systemic Delivery of Aptamer-Conjugated XBP1 SiRNA Nanoparticles for Efficient Suppression of HER2+ Breast Cancer. ACS Appl. Mater. Interfaces.

[118] Rybakova Y., Kowalski P.S., Huang Y., Gonzalez J.T., Heartlein M.W., DeRosa F., Delcassian D., Anderson D.G. (2019). MRNA Delivery for Therapeutic Anti-HER2 Antibody Expression In Vivo. Mol. Ther..

[119] Cai Z., Yook S., Lu Y., Bergstrom D., Winnik M.A., Pignol J.-P., Reilly R.M. (2017). Local Radiation Treatment of HER2-Positive Breast Cancer Using Trastuzumab-Modified Gold Nanoparticles Labeled with 177Lu. Pharm. Res..

[120] Guryev E.L., Volodina N.O., Shilyagina N.Y., Gudkov S.V., Balalaeva I.V., Volovetskiy A.B., Lyubeshkin A.V., Sen’ A.V., Ermilov S.A., Vodeneev V.A. (2018). Radioactive (^90^ Y) Upconversion Nanoparticles Conjugated with Recombinant Targeted Toxin for Synergistic Nanotheranostics of Cancer. Proc. Natl. Acad. Sci. USA.

[121] Mironova K.E., Khochenkov D.A., Generalova A.N., Rocheva V.V., Sholina N.V., Nechaev A.V., Semchishen V.A., Deyev S.M., Zvyagin A.V., Khaydukov E.V. (2017). Ultraviolet Phototoxicity of Upconversion Nanoparticles Illuminated with Near-Infrared Light. Nanoscale.

[122] Aman N.A., Doukoure B., Koffi K.D., Koui B.S., Traore Z.C., Kouyate M., Effi A.B. (2019). HER2 Overexpression and Correlation with Other Significant Clinicopathologic Parameters in Ivorian Breast Cancer Women. BMC Clin. Pathol..

[123] Vi C., Mandarano G., Shigdar S. (2021). Diagnostics and Therapeutics in Targeting HER2 Breast Cancer: A Novel Approach. Int. J. Mol. Sci..

[124] Gutierrez C., Schiff R. (2011). HER2: Biology, Detection, and Clinical Implications. Arch. Pathol. Lab. Med..

[125] Salahandish R., Ghaffarinejad A., Naghib S.M., Majidzadeh-A K., Zargartalebi H., Sanati-Nezhad A. (2018). Nano-Biosensor for Highly Sensitive Detection of HER2 Positive Breast Cancer. Biosens. Bioelectron..

[126] Phillips K.A., Marshall D.A., Haas J.S., Elkin E.B., Liang S.-Y., Hassett M.J., Ferrusi I., Brock J.E., Van Bebber S.L. (2009). Clinical Practice Patterns and Cost Effectiveness of Human Epidermal Growth Receptor 2 Testing Strategies in Breast Cancer Patients. Cancer.

[127] Liu M., Yu X., Chen Z., Yang T., Yang D., Liu Q., Du K., Li B., Wang Z., Li S. (2017). Aptamer Selection and Applications for Breast Cancer Diagnostics and Therapy. J. Nanobiotechnol..

[128] Mathenge E.G., Dean C.A., Clements D., Vaghar-Kashani A., Photopoulos S., Coyle K.M., Giacomantonio M., Malueth B., Nunokawa A., Jordan J. (2014). Core Needle Biopsy of Breast Cancer Tumors Increases Distant Metastases in a Mouse Model. Neoplasia.

[129] Castro-Giner F., Aceto N. (2020). Tracking Cancer Progression: From Circulating Tumor Cells to Metastasis. Genome Med..

[130] Chen W., Li X., Zhu L., Liu J., Xu W., Wang P. (2017). Preclinical and Clinical Applications of Specific Molecular Imaging for HER2-Positive Breast Cancer. Cancer Biol. Med..

[131] Gao D., Gao J., Xu M., Cao Z., Zhou L., Li Y., Xie X., Jiang Q., Wang W., Liu J. (2017). Targeted Ultrasound-Triggered Phase Transition Nanodroplets for Her2-Overexpressing Breast Cancer Diagnosis and Gene Transfection. Mol. Pharm..

[132] Busquets M.A., Estelrich J., Sánchez-Martín M.J. (2015). Nanoparticles in Magnetic Resonance Imaging: From Simple to Dual Contrast Agents. Int. J. Nanomed..

[133] Chen Q., Xu L., Liang C., Wang C., Peng R., Liu Z. (2016). Photothermal Therapy with Immune-Adjuvant Nanoparticles Together with Checkpoint Blockade for Effective Cancer Immunotherapy. Nat. Commun..

[134] Pellico J., Llop J., Fernández-Barahona I., Bhavesh R., Ruiz-Cabello J., Herranz F. (2017). Iron Oxide Nanoradiomaterials: Combining Nanoscale Properties with Radioisotopes for Enhanced Molecular Imaging. Contrast Media Mol. Imaging.

[135] Chen T.-J., Cheng T.-H., Chen C.-Y., Hsu S.C.N., Cheng T.-L., Liu G.-C., Wang Y.-M. (2009). Targeted Herceptin–Dextran Iron Oxide Nanoparticles for Noninvasive Imaging of HER2/Neu Receptors Using MRI. J. Biol. Inorg. Chem..

[136] Alric C., Hervé-Aubert K., Aubrey N., Melouk S., Lajoie L., Même W., Même S., Courbebaisse Y., Ignatova A.A., Feofanov A.V. (2018). Targeting HER2-Breast Tumors with ScFv-Decorated Bimodal Nanoprobes. J. Nanobiotechnol..

[137] Zhang Y., Ni Q., Xu C., Wan B., Geng Y., Zheng G., Yang Z., Tao J., Zhao Y., Wen J. (2019). Smart Bacterial Magnetic Nanoparticles for Tumor-Targeting Magnetic Resonance Imaging of HER2-Positive Breast Cancers. ACS Appl. Mater. Interfaces.

[138] Lim E.-K., Kim T., Paik S., Haam S., Huh Y.-M., Lee K. (2015). Nanomaterials for Theranostics: Recent Advances and Future Challenges. Chem. Rev..

[139] Hardman R. (2006). A Toxicologic Review of Quantum Dots: Toxicity Depends on Physicochemical and Environmental Factors. Environ. Health Perspect..

[140] Wei T., Xing H., Wang H., Zhang Y., Wang J., Shen J., Dai Z. (2020). Bovine Serum Albumin Encapsulation of near Infrared Fluorescent Nano-Probe with Low Nonspecificity and Cytotoxicity for Imaging of HER2-Positive Breast Cancer Cells. Talanta.

[141] Seifalian A., Rizvi S., Rouhi S., Taniguchi S., Yang S.Y., Green M., Keshtgar M. (2014). Near-Infrared Quantum Dots for HER2 Localization and Imaging of Cancer Cells. Int. J. Nanomed..

[142] Wang Z., Wang W., Bu X., Wei Z., Geng L., Wu Y., Dong C., Li L., Zhang D., Yang S. (2015). Microarray Based Screening of Peptide Nano Probes for HER2 Positive Tumor. Anal. Chem..

[143] Ramos-Gomes F., Bode J., Sukhanova A., Bozrova S.V., Saccomano M., Mitkovski M., Krueger J.E., Wege A.K., Stuehmer W., Samokhvalov P.S. (2018). Single- and Two-Photon Imaging of Human Micrometastases and Disseminated Tumour Cells with Conjugates of Nanobodies and Quantum Dots. Sci. Rep..

[144] Xavier C., Blykers A., Vaneycken I., D’Huyvetter M., Heemskerk J., Lahoutte T., Devoogdt N., Caveliers V. (2016). 18F-Nanobody for PET Imaging of HER2 Overexpressing Tumors. Nucl. Med. Biol..

[145] Ahlgren S., Wållberg H., Tran T.A., Widström C., Hjertman M., Abrahmsén L., Berndorff D., Dinkelborg L.M., Cyr J.E., Feldwisch J. (2009). Targeting of HER2-Expressing Tumors with a Site-Specifically 99m Tc-Labeled Recombinant Affibody Molecule, ZHER2: 2395, with C-Terminally Engineered Cysteine. J. Nucl Med..

[146] Welch M.J., Hawker C.J., Wooley K.L. (2009). The Advantages of Nanoparticles for PET. J. Nucl Med..

[147] Lee H., Shields A.F., Siegel B.A., Miller K.D., Krop I., Ma C.X., LoRusso P.M., Munster P.N., Campbell K., Gaddy D.F. (2017). 64Cu-MM-302 Positron Emission Tomography Quantifies Variability of Enhanced Permeability and Retention of Nanoparticles in Relation to Treatment Response in Patients with Metastatic Breast Cancer. Clin. Cancer Res..

[148] Rainone P., Riva B., Belloli S., Sudati F., Ripamonti M., Verderio P., Colombo M., Colzani B., Gilardi M.C., Moresco R.M. (2017). Development of 99mTc-Radiolabeled Nanosilica for Targeted Detection of HER2-Positive Breast Cancer. Int. J. Nanomed..

[149] Lee H., Zheng J., Gaddy D., Orcutt K.D., Leonard S., Geretti E., Hesterman J., Harwell C., Hoppin J., Jaffray D.A. (2015). A Gradient-Loadable 64Cu-Chelator for Quantifying Tumor Deposition Kinetics of Nanoliposomal Therapeutics by Positron Emission Tomography. Nanomed. Nanotechnol. Biol. Med..

[150] Rainone P., De Palma A., Sudati F., Roffia V., Rigamonti V., Salvioni L., Colombo M., Ripamonti M., Spinelli A.E., Mazza D. (2021). 99mTc-Radiolabeled Silica Nanocarriers for Targeted Detection and Treatment of HER2-Positive Breast Cancer. Int. J. Nanomed..

[151] Jang M., Yoon Y.I., Kwon Y.S., Yoon T.-J., Lee H.J., Hwang S.I., Yun B.L., Kim S.M. (2014). Trastuzumab-Conjugated Liposome-Coated Fluorescent Magnetic Nanoparticles to Target Breast Cancer. Korean J. Radiol..

[152] Jiang Q., Hao S., Xiao X., Yao J., Ou B., Zhao Z., Liu F., Pan X., Luo B., Zhi H. (2016). Production and Characterization of a Novel Long-Acting Herceptin-Targeted Nanobubble Contrast Agent Specific for Her-2-Positive Breast Cancers. Breast Cancer.

[153] Li X., Xia S., Zhou W., Ji R., Zhan W. (2019). Targeted Fe-Doped Silica Nanoparticles as a Novel Ultrasound–Magnetic Resonance Dual-Mode Imaging Contrast Agent for HER2-Positive Breast Cancer. Int. J. Nanomed..

[154] Chen J.S., Chen J., Bhattacharjee S., Cao Z., Wang H., Swanson S.D., Zong H., Baker J.R., Wang S.H. (2020). Functionalized Nanoparticles with Targeted Antibody to Enhance Imaging of Breast Cancer in Vivo. J. Nanobiotechnol..

[155] Chen F., Ma K., Madajewski B., Zhuang L., Zhang L., Rickert K., Marelli M., Yoo B., Turker M.Z., Overholtzer M. (2018). Ultrasmall Targeted Nanoparticles with Engineered Antibody Fragments for Imaging Detection of HER2-Overexpressing Breast Cancer. Nat. Commun..

[156] Wu Y., Meng Q., Yang Z., Shi L., Hu R., Zhang P., Wei J., Ren J., Leng B., Xu D. (2018). Circulating HER-2 MRNA in the Peripheral Blood as a Potential Diagnostic and Prognostic Biomarker in Females with Breast Cancer. Oncol. Lett..

[157] Emami M., Shamsipur M., Saber R., Irajirad R. (2014). An Electrochemical Immunosensor for Detection of a Breast Cancer Biomarker Based on AntiHER2–Iron Oxide Nanoparticle Bioconjugates. Analyst.

[158] Villegas-Serralta E., Zavala O., Flores-Urquizo I.A., García-Casillas P.E., Chapa González C. (2018). Detection of HER2 through Antibody Immobilization Is Influenced by the Properties of the Magnetite Nanoparticle Coating. J. Nanomater..

[159] Yang J., Wang Z., Zong S., Song C., Zhang R., Cui Y. (2012). Distinguishing Breast Cancer Cells Using Surface-Enhanced Raman Scattering. Anal. Bioanal. Chem..

[160] Tao Y., Li M., Kim B., Auguste D.T. (2017). Incorporating Gold Nanoclusters and Target-Directed Liposomes as a Synergistic Amplified Colorimetric Sensor for HER2-Positive Breast Cancer Cell Detection. Theranostics.

[161] Tabatabaei-Panah A.-S., Jeddi-Tehrani M., Ghods R., Akhondi M.-M., Mojtabavi N., Mahmoudi A.-R., Mirzadegan E., Shojaeian S., Zarnani A.-H. (2013). Accurate Sensitivity of Quantum Dots for Detection of HER2 Expression in Breast Cancer Cells and Tissues. J. Fluoresc..

[162] Gijs M., Penner G., Blackler G., Impens N., Baatout S., Luxen A., Aerts A. (2016). Improved Aptamers for the Diagnosis and Potential Treatment of HER2-Positive Cancer. Pharmaceuticals.

[163] Chu M., Kang J., Wang W., Li H., Feng J., Chu Z., Zhang M., Xu L., Wang Y. (2017). Evaluation of Human Epidermal Growth Factor Receptor 2 in Breast Cancer with a Novel Specific Aptamer. Cell. Mol. Immunol..

[164] Kim J.-H., Suh J.-S., Yang J. (2020). Labeling-Free Detection of ECD-HER2 Protein Using Aptamer-Based Nano-Plasmonic Sensor. Nanotechnology.

[165] Zheng D., Wan C., Yang H., Xu L., Dong Q., Du C., Du J., Li F. (2020). Her2-Targeted Multifunctional Nano-Theranostic Platform Mediates Tumor Microenvironment Remodeling and Immune Activation for Breast Cancer Treatment. Int. J. Nanomed..

[166] Choi W.I., Lee J.H., Kim J.-Y., Heo S.U., Jeong Y.Y., Kim Y.H., Tae G. (2015). Targeted Antitumor Efficacy and Imaging via Multifunctional Nano-Carrier Conjugated with Anti-HER2 Trastuzumab. Nanomed. Nanotechnol. Biol. Med..

